# Formate Metabolism in Shigella flexneri and Its Effect on HeLa Cells at Different Stages during the Infectious Process

**DOI:** 10.1128/spectrum.00631-22

**Published:** 2023-04-12

**Authors:** Ke-Chuan Wang, Mathilde Hauge Lerche, Jan Henrik Ardenkjær-Larsen, Pernille Rose Jensen

**Affiliations:** a Center for Hyperpolarization in Magnetic Resonance, Department of Health Technology, Technical University of Denmark, Kongens Lyngby, Denmark; Forschungszentrum Jülich GmbH

**Keywords:** *Shigella*, formate, host-pathogen interactions, metabolism

## Abstract

Shigellosis caused by *Shigella* is one of the most important foodborne illnesses in global health, but little is known about the metabolic cross talk between this bacterial pathogen and its host cells during the different stages of the infection process. A detailed understanding of the metabolism can potentially lead to new drug targets remedying the pressing problem of antibiotic resistance. Here, we use stable isotope-resolved metabolomics as an unbiased and fast method to investigate how *Shigella* metabolizes ^13^C-glucose in three different environments: inside the host cells, adhering to the host cells, and alone in suspension. We find that especially formate metabolism by bacteria is sensitive to these different environments. The role of formate in pathogen metabolism is sparsely described in the literature compared to the roles of acetate and butyrate. However, its metabolic pathway is regarded as a potential drug target due to its production in microorganisms and its absence in humans. Our study provides new knowledge about the regulatory effect of formate. Bacterial metabolism of formate is pH dependent when studied alone in culture medium, whereas this effect is less pronounced when the bacteria adhere to the host cells. Once the bacteria are inside the host cells, we find that formate accumulation is reduced. Formate also affects the host cells resulting in a reduced infection rate. This was correlated to an increased immune response. Thus, intriguingly formate plays a double role in pathogenesis by increasing the virulence of *Shigella* and at the same time stimulating the immune response of the host.

**IMPORTANCE** Bacterial infection is a pressing societal concern due to development of resistance toward known antibiotics. Central carbon metabolism has been suggested as a potential new target for drug development, but metabolic changes upon infection remain incompletely understood. Here, we used a cellular infection model to study how the bacterial pathogen *Shigella* adapts its metabolism depending on the environment starting from the extracellular medium until *Shigella* successfully invaded and proliferated inside host cells. The mixed-acid fermentation of *Shigella* was the major metabolic pathway during the infectious process, and the glucose-derived metabolite formate surprisingly played a divergent role in the pathogen and in the host cell. Our data show reduced infection rate when both host cells and bacteria were treated with formate, which correlated with an upregulated immune response in the host cells. The formate metabolism in *Shigella* thus potentially provides a route toward alternative treatment strategies for *Shigella* prevention.

## INTRODUCTION

Treatment of bacterial infection with the development of antibiotic resistance is an urgent societal concern (1), and this concern is further enhanced by the slow development of new types of antibiotics (2). Central carbon metabolism has been identified as an underexplored antimicrobial drug target (3). Most antimicrobial drugs target bacterial cell-wall or protein synthesis, but the low permeability across the host cell membrane and bacteria-containing vacuoles reduces the efficacy of some kinds of antibiotics toward intracellular pathogens (4, 5). Central carbon metabolism, conversely, must be sustained by the pathogens for energy and redox balance. To exploit the potential of targeting central carbon metabolism, a detailed understanding of the bacterial metabolism is paramount. But, how pathogens sense and adapt their metabolism when changing the environment from extra- to intracellular is largely unknown (6).

Shigellosis caused by the bacterial pathogen *Shigella* is one of the most important foodborne illnesses in global health, and its intracellular proliferation within the human host is the most crucial step for inducing shigellosis (7). A global proteome analysis identified that mixed-acid fermentation, and the metabolism of pyruvate, in particular, was required for optimal intracellular growth of Shigella flexneri (8). Another study confirmed the importance of pyruvate for internalized *Shigella* in a human host. It was shown that *Shigella* rerouted the central carbon metabolism of the host cells by capturing the high energy metabolite pyruvate and metabolizing it to acetate (9). Formate has also been shown to promote *Shigella* intercellular spread and virulence (6). Proteome analysis found that the formate transporter FocA was not expressed during intracellular growth and that it was 5-fold increased during bacterial growth in the presence of human host cells compared to *Shigella* grown free in suspension (8). In the same study, pyruvate formate-lyase (PflB) and alcohol dehydrogenase (AdhE) were also increased during growth in the presence of human host cells compared to bacteria grown free in suspension.

Formate, one of the short-chain fatty acids (SCFAs), is produced as a result of the digestive processes by the intestinal microbiota. Its concentration is around 10 mM in the gastrointestinal tract in the first year of infants, whereafter it decreases (10). Up to 95% of total SCFAs in the adult consists of acetate, propionate, and butyrate in a 3:1:1 ratio (11). The SCFA concentrations vary ranging from 20 mM to 40 mM in the small intestine and can be higher than 100 mM in the colon (12). In humans, the function of formate is mainly coupled to utilization of folate and vitamin B_12_ (13). Acetate and formate are products of the mixed-acid fermentation taking place in bacterial primary fermenters, to which pathogens like *Shigella*, Escherichia coli, and *Salmonella* belong, whereas propionate and butyrate are produced by bacterial secondary fermenters (14). There is, thus, a distinction between which bacteria produce acetate and formate compared to propionate and butyrate, and their biological role has also been reported to be different. From studies on *Salmonella*, acetate and formate have been demonstrated to induce invasion genes while propionate and butyrate repress them (12, 15–17). It has been shown that *Shigella* pathology in patients was improved by receiving butyrate-containing enemas compared to a placebo group (18). Manipulation of SCFA levels, thus, have prospects for improving the outcome of *Shigella* pathogenesis. The mechanism is still unclear, but it might include protective stimulation of the host defense mechanisms (14).

Here, we used stable isotope-resolved metabolomics (SIRM) nuclear magnetic resonance (NMR) as a sensitive method to untangle the central carbon metabolism of the bacterial pathogen *Shigella* from the host cell. In one assay platform, the three major steps in pathogen infection metabolism could be measured as follows: pathogen and noninfected host cells separately, pathogen adhering to host cells, and pathogen-infected host cells. Within this assay platform, we investigated the effect of SCFAs both on bacterial virulence and on host defense. This double-sided approach revealed formate as an active metabolite in pathogenesis, which both increased bacterial virulence and, at the same time, increased the immune response in the host cell.

## RESULTS

### Noninvasive direct measurements of metabolism during the infection process.

**FIG 1:**
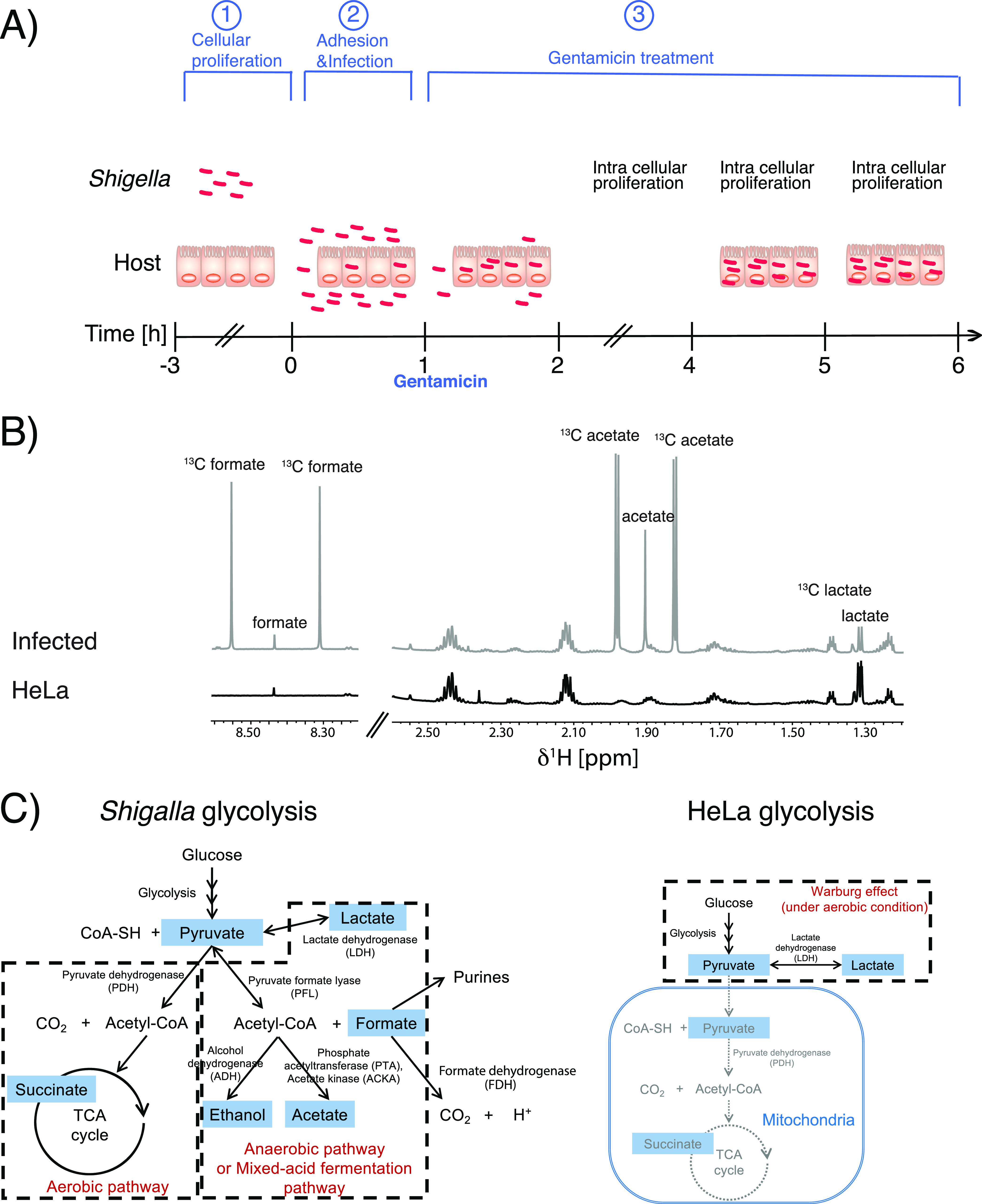
The *Shigella* infection model for the metabolic analysis by ^1^H NMR. (A) A three-step infection model. In the first part, *Shigella* is alone in suspension where it is cultured to mid-log for 3 h. HeLa is likewise separately cultured. In the second part, HeLa is infected with mid-log cultured *Shigella* (MOI, 100). In the third part, after an initial 1-h infection, HeLa cells are treated with 100 μg/mL gentamicin which kills the extracellular bacteria. (B) Part of ^1^H NMR spectrum obtained directly on the cell medium. The typical signal pattern produced by metabolites downstream of [U-^13^C] glucose 6-h after *Shigella* infection is highlighted. (C) The metabolic pathway of glucose for *Shigella* and HeLa cells showing how *Shigella* can utilize both aerobic and anaerobic metabolism, and HeLa cells mainly utilize aerobic metabolism.

The intracellular pathogen infection process was studied through the actions of the bacterial pathogen Shigella flexneri. To be able to separately study the metabolism in all stages during the infection process, an assay was constructed that simulated three distinct environments of the pathogen (Fig. 1A). In the first part of the assay, *Shigella* proliferated freely much like in the gut where it competes with all of the other microbes in the microbiome. In the second part, *Shigella* was brought in contact with the host cells (HeLa) to which it adhered similarly to how it would adhere to the gut epithelial cells. In this part of the assay, *Shigella* was also internalized. Once inside the host cell in the third and last part of the assay, *Shigella* proliferated inside the HeLa cells, where it acted in a compartment restricted to the nutrients taken up by the host cell. In this three-part assay, we could study *Shigella* metabolism in one coherent assay from alone in suspension, adhered to its host, and inside its host. The metabolism of the infected and noninfected HeLa cells was followed without change of medium for the full time period. This had the benefit of minimal sample handling and allowed tracking of the ^13^C-metabolic output without any estimations, but it resulted in different types of metabolism dominating at different time points of the assay (Fig. 1A).

We used ^1^H NMR as a simple powerful tool to noninvasively study the metabolism of *Shigella* during these different steps of the infection process. Stable isotope-labeled [U-^13^C]-glucose was used to highlight the metabolites from glycolysis in the background of media components. Examples of ^1^H NMR spectra from infected and noninfected HeLa cells at the endpoint of the infection period (6-h postinfection [p.i.]) are shown in Fig. 1B. End products of glycolysis are highlighted by the easily recognized signal pattern caused by ^13^C-^1^H interaction. The bacterial mixed-acid fermentation produces five main metabolites as follows: acetate, formate, ethanol, lactate, and succinate. Out of these, acetate, formate, and ethanol provide excellent biomarkers for the bacterial metabolism since little or none are produced by the host cells (Fig. 1C).

**FIG 2:**
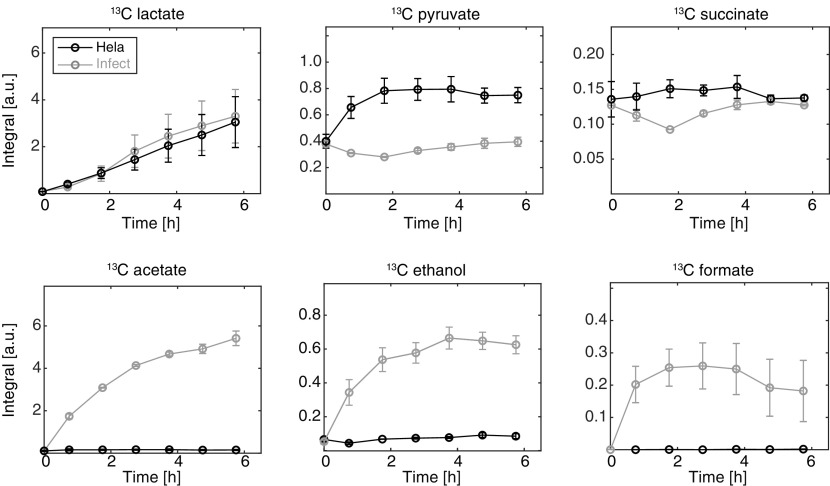
Time course of extracellular ^13^C-labeled metabolites from HeLa cells infected with *Shigella*. The metabolism of [U-^13^C] glucose was followed in infected (infect) and noninfected HeLa cells (HeLa) (*n* = 2). Each sample was made according to the procedure of the continuous measurement of metabolism (see Fig. S1A in the supplemental material). Briefly, 100 μg/mL gentamicin was added at 1 h p.i. and was kept without medium replacement during the total 6-h incubation. The ^13^C-labeled metabolites are obtained from the symmetric ^13^C-satellites displaying the characteristic ^1^J_(13C,1H)_ coupling of 130 Hz in the 1D ^1^H NMR spectra.

The noninfected HeLa cells mainly produced ^13^C-lactate with a linear rate throughout the full infection process (Fig. 2). ^13^C-pyruvate reached a steady state 2 h p.i., whereas the tricarboxylic acid (TCA) cycle intermediate ^13^C-succinate did not show any change over the time course of the assay. ^13^C-acetate, ^13^C-ethanol, and ^13^C-formate were, as expected, not produced by the HeLa cells. For the infected HeLa cells, ^13^C-lactate was produced with a linear rate throughout the assay. The rate was equal to the noninfected HeLa cells, and thus, the glycolytic rate of the host cells was not affected by *Shigella* or gentamicin treatment. The metabolites ^13^C-acetate, ^13^C-ethanol, and ^13^C-formate were observed when *Shigella* was introduced. Within the first hour of infection (0 to 1 h p.i.), ^13^C-acetate, ^13^C-ethanol, and ^13^C-formate were formed. In this period, the vast majority of the bacteria were extracellular or adhered to the host cells. The antibiotic gentamicin was added 1 h p.i. The metabolite ^13^C-acetate continued to build up throughout the rest of the assay period (1 to 6 h p.i.), while ^13^C-ethanol increased toward a saturating plateau. On the contrary, the accumulation of ^13^C-formate did not increase after the addition of gentamicin. The full ^1^H NMR spectrum to the time 6 h p.i. was used as input for a discrimination between infected and noninfected host cells at the end of the infection period (see Fig. S2 in the supplemental material). From this, a statistical principal-component analysis (PCA) showed that the three metabolites acetate, ethanol, and formate were the main metabolites responsible for differentiating between the noninfected and infected HeLa cells 6 h p.i.

### Formate accumulation is decreased during intracellular proliferation.

**FIG 3:**
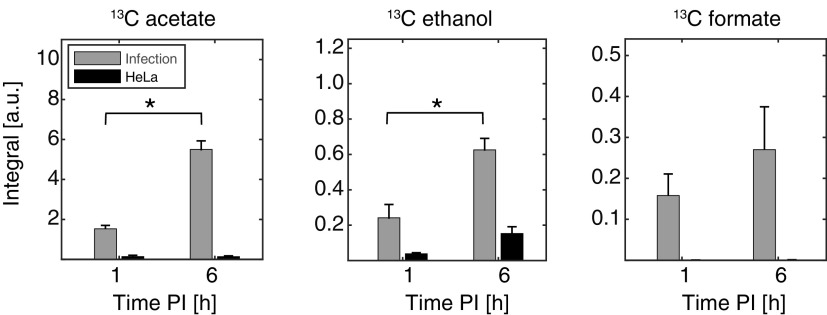
Comparison of the production of extracellular ^13^C-metabolites 1 h p.i. and up to 6 h p.i. from HeLa cells and *Shigella*-infected HeLa cells as measured by ^1^H NMR. Each sample was made according to the procedure of the continuous measurement of metabolism (see Fig. S1A in the supplemental material). Briefly, 100 μg/mL gentamicin was added at 1 h p.i. and was kept without medium replacement during the total 6-h incubation. Each sample was saved at 1 h p.i. and 6 h p.i. and was analyzed by ^1^H NMR. The statistical analysis was done by the comparison of the ^13^C-metabolites produced between 1 h p.i. and 6 h p.i. All data are represented as mean ± standard error of the mean (SEM), and an asterisk indicated a statistical difference (*P *≤ 0.05); *n* = 4.

**FIG 4:**
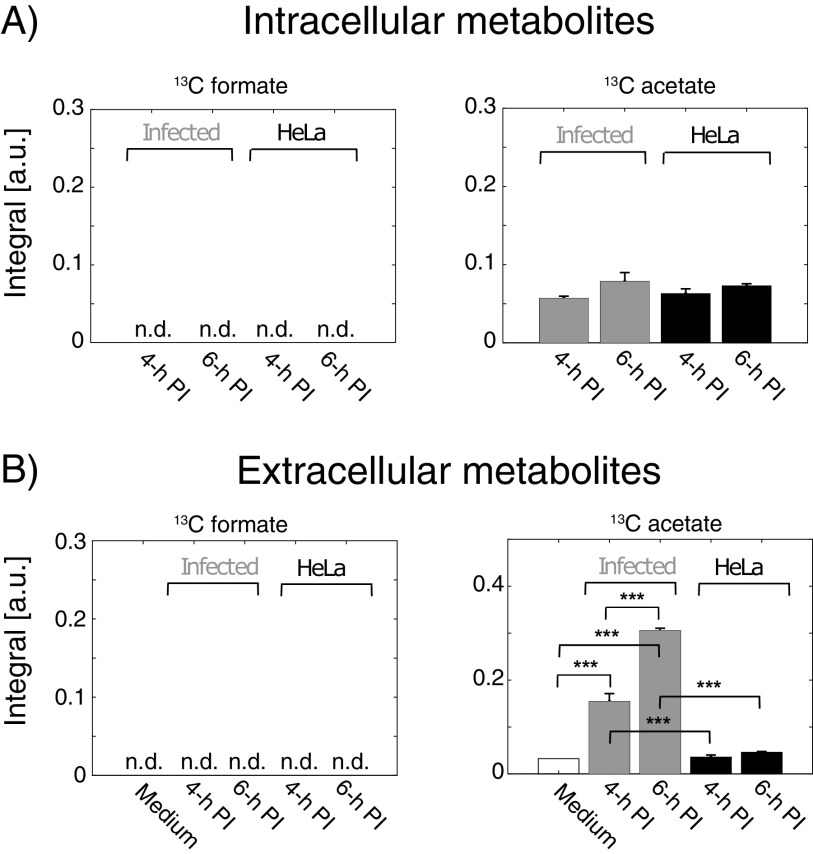
^1^H NMR analysis of ^13^C-metabolites produced from HeLa cells and *Shigella*-infected HeLa cells. The analyses of both extracellular and intracellular samples were performed according to the procedure of metabolism from intracellular bacteria (see Fig. S1B in the supplemental material). Briefly, after 1 h treatment with 100 μg/mL gentamicin, each medium was replaced with fresh medium containing 10 μg/mL of gentamicin for an additional 4-h incubation. At 4 h and 6 h p.i., the supernatants and samples of PCA extracted cell lysates were analyzed by ^1^H NMR. (A) The intracellular metabolites from the PCA extracted cells at 4 h and 6 h p.i. No ^13^C-formate could be detected (see Fig. S3 in the supplemental material for the NMR spectrum). (B) The extracellular supernatants collected at 4 h and 6 h p.i. showed no detection of ^13^C-formate. A significant production between 4 h and 6 h p.i. was determined for ^13^C-acetate in the infected group, whereas no significant production was measured for HeLa cells. Asterisks indicate the statistical difference (***, *P* ≤ 0.001) by ANOVA. Data are represented as mean ± SEM (*n* = 3).

To separate the metabolism generated from extracellular bacteria in the first part of the infection process from the metabolism produced by intracellular bacteria in the last part of the process, a comparison of the metabolic profiles between 1 h p.i. and 6 h p.i. was made (Fig. 3). From this comparison, it was observed that acetate and ethanol were produced by the intracellular *Shigella* (*P *≤ 0.05), whereas only an increasing tendency, which was not significant, was found for formate. That formate metabolism was affected by bacteria changing from extracellular to the intracellular environment was further investigated. Metabolism from infected and noninfected HeLa cells was examined by analyzing the extracellular media and the corresponding intracellular metabolites. The latter were obtained by perchloric acid extraction. Thus, due to different sample preparation protocols, the intracellular and extracellular metabolite assays cannot be directly compared (Fig. 4). Neither in the infected nor in the noninfected HeLa cells was it possible to detect intracellular ^13^C-formate. In the same assay, intracellular ^13^C-acetate was detected and a tendency to produce ^13^C-acetate between 4 and 6 h p.i. for both groups was observed although below statistical significance (Fig. 4A). Amounts of extracellular metabolites were analyzed in the supernatants from the same infection samples. Again, no ^13^C-formate could be detected while ^13^C-acetate significantly increased between 4 and 6 h for the infected group, and no significant production was found in the noninfected group (Fig. 4B). Using a set of reference samples with ^13^C-acetate and ^13^C-formate in concentrations from 0 to 100 μM, the quantification limit for the ^1^H NMR protocol was determined to be 5 μM for both metabolites (see Fig. S4 in the supplemental material). The extracellular ratio ^13^C-acetate/^13^C-formate was determined to be >10 for the infected HeLa cells at both 4 h and 6 h p.i. Second, the protocol for the gentamicin assay was scaled up to 10 million HeLa cells. At 2 h p.i., the cells were harvested by trypsinization and incubated with [U-^13^C] glucose for 2 h (2 to 4 h p.i.) (see Fig. S1C in the supplemental material). The total (extracellular plus intracellular) metabolite content was extracted with perchloric acid. A sensitivity enhanced NMR method (dissolution Dynamic Nuclear Polarization [dDNP-NMR]) was applied to analyze the samples. In contrast to ^1^H NMR, this method is sparse in signals and directly detects ^13^C-isotope-labeled metabolites (19). This assay showed, in accordance with the previous measurements (Fig. 4), increased acetate production (*P = *0.03) and no change in formate accumulation (see Fig. S5 in the supplemental material). That no formate metabolism could be detected in either of these assays was taken as evidence for reduced formate accumulation relative to acetate when *Shigella* proliferated intracellularly.

### Role of formate metabolism for *Shigella* virulence.

**FIG 5:**
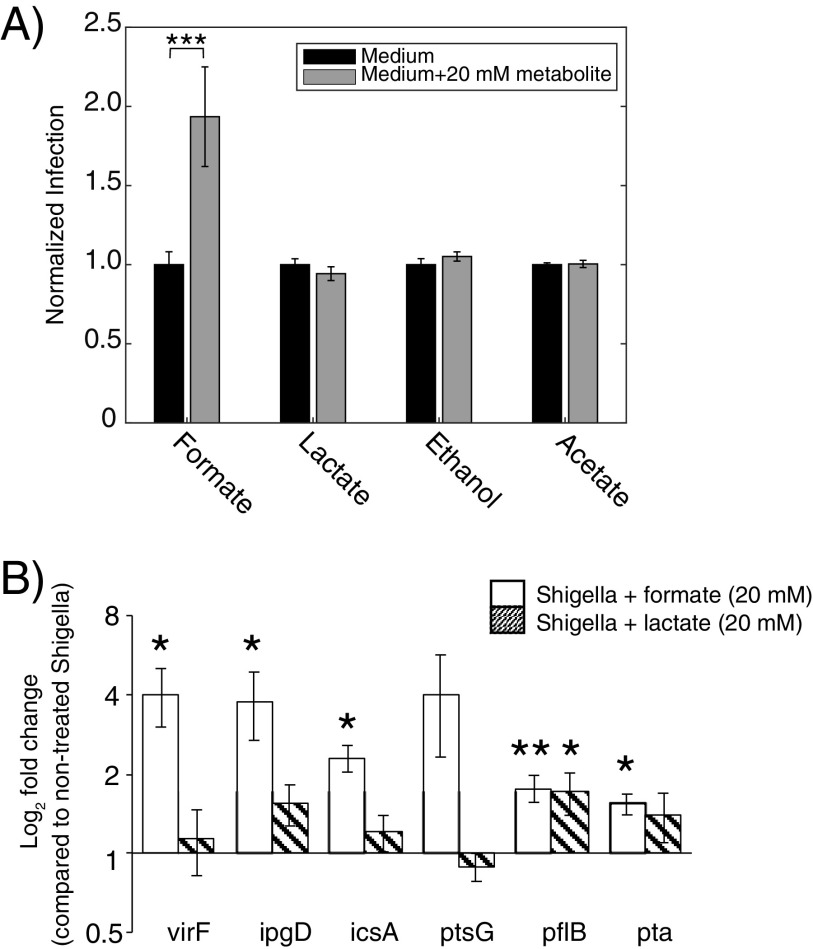
The role of metabolites from bacterial mixed-acid fermentation on *Shigella* virulence. (A) *Shigella* was treated with 20 mM metabolites formate, lactate, ethanol, and acetate for 3 h before infection (or not). The treatment hereafter continued in the additional 6-h adhesion and intracellular proliferation period. Each experiment was performed in triplicate. (B) Six selected genes related to virulence and glycolysis were analyzed with qRT-PCR. The nontreated group relative to the 20 mM formate-treated (open bars) group and the nontreated group relative to the 20 mM lactate-treated (slash bars) group were analyzed individually. The expression level of each mRNA was calculated according to the ΔΔ*C_T_* method. Data was measured in triplicate. All data are represented as mean ± SEM, and asterisks indicated a statistical difference as follows: *, *P* ≤ 0.05; **, *P* ≤ 0.01; and ***, *P* ≤ 0.001 compared to the nontreated group.

Metabolites from bacterial mixed-acid fermentation were investigated for their antimicrobial effect to understand how formate metabolism from internalized *Shigella* could be coupled to the infection process. The four abundant metabolites, acetate, formate, lactate, and ethanol, were administered in concentrations (20 mM) mimicking the SCFA gut concentrations during infection of HeLa cells with *Shigella*. A 2-fold increase in internalized *Shigella* was measured when *Shigella* had been exposed to 20 mM formate during the free proliferation period ([−3] to 0 h p.i.) (Fig. 1A), whereas no change was observed with a similar treatment of *Shigella* with lactate, ethanol, or acetate (Fig. 5A). We then analyzed virulence and central carbon metabolism-related genes from *Shigella*, which potentially could be affected by the metabolites (i.e., virulence regulon transcriptional activator *virF*, inositol phosphate phosphatase *ipgD*, autotransporter protein *icsA*, glucose transporter subunit *ptsG*, pyruvate formate-lyase *pflB*, and phosphate acetyltransferase *pta*). The genes were selected among major known indicators involved in pathogen infection (see Table S2 in the supplemental material). *Shigella* was treated with 20 mM formate or lactate during the free proliferation period, and the gene expressions were analyzed with quantitative real-time reverse transcriptase-PCR (qRT-PCR) using 16s rRNA as internal control. The virulence related genes *virF*, *ipgD*, and *icsA* were significantly upregulated by formate compared to the nontreated group, whereas no significant changes were measured when *Shigella* was treated with lactate (Fig. 5B). Also, a significantly raised mRNA expression of the glycolytic genes, *pflB* and *pta*, was observed in the formate-treated *Shigella*, and lactate pretreatment significantly enhanced *pflB* expression as well. Thus, formate availability increased *Shigella* infection and partially mediated immune regulations as evidenced respectively by the number of internalized *Shigella* as well as enhanced gene expression of key metabolic and virulence genes.

### Fluctuating formate metabolism in different stages of the infection process.

**FIG 6:**
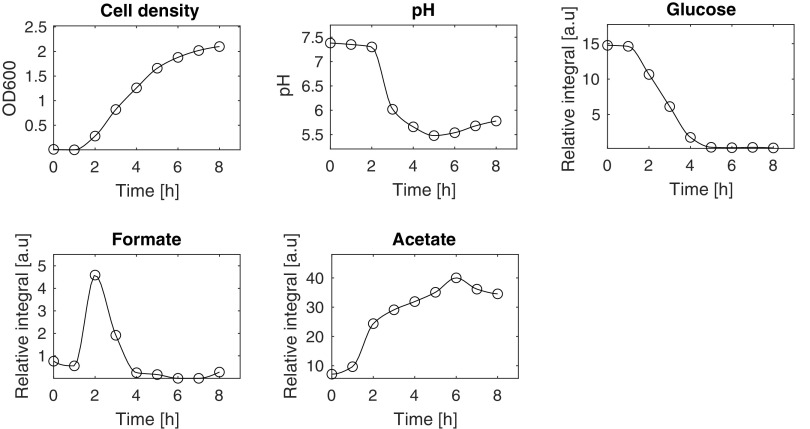
Profiles of cell density, pH, and the consumption and production of metabolites during *Shigella* growth. *Shigella* was grown in TSB medium at 37°C with shaking at 200 rpm for 8 h. Cell density and pH were measured on a supernatant sample taken every hour, and metabolic profiles were measured on the same sample with ^1^H NMR.

To get further insight into how formate metabolism could play a role in the infection, we investigated its metabolic profile in the different stages of the process. First, *Shigella* growth was investigated in suspension culture by evaluating cell density, pH, and consumption of glucose as well as production of acetate and formate (Fig. 6). *Shigella* reached mid-log stage after around 3 h growth. The pH of the medium decreased from 7.4 to 6.0 in this period. After 3 h, when the culture was at the mid-log stage, about 50% of the glucose was still present. The production of acetate followed the growth of the cells, whereas formate showed a temporary increase until 2 h followed by a steep decline. These data showed that formate metabolism was very sensitive to the growth conditions. In the exponential phase, formate was produced until pH drops to 6. Since glucose availability was high at this point, it was plausible that formate metabolism was mainly pH dependent at this stage. Such pH-dependent metabolic profile for formate is in accordance with previous studies of Escherichia coli, a close relative to *Shigella*, studied under similar conditions to ours (20). For E. coli, the reduced formate accumulation was explained by a strong pH dependency of the formate transporter FocA, which switches mechanism around pH 5.5.

**FIG 7:**
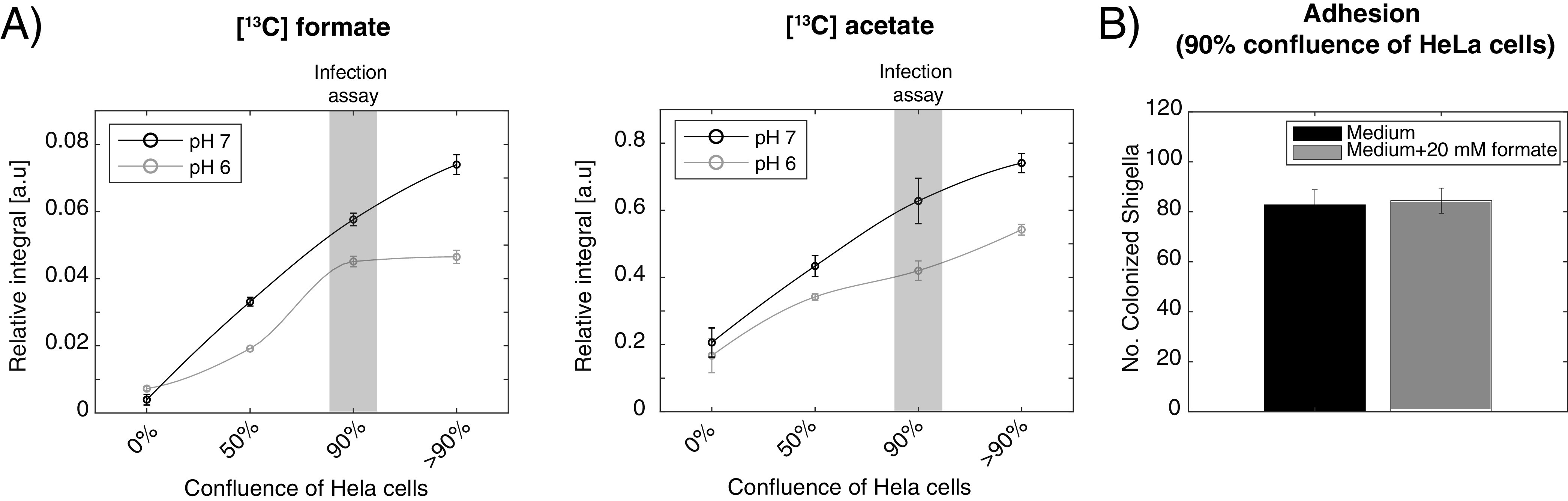
Impact of pH on *Shigella* metabolism in the presence of HeLa cells. (A) pH dependence of ^13^C-formate and ^13^C-acetate production as function of host cell density. Extracellular metabolite production from [U-^13^C] glucose was measured with ^1^H NMR 1 h after addition of the same number of *Shigella* (5 × 10^7^) to varying density of HeLa cells (0, 50, 90, and >90% corresponding to 0, 0.25, 0.5, or 1 million). For each cell concentration, the growth medium had been adjusted to either pH 6 or 7 prior to the addition of *Shigella*. Gray box indicates the conditions used for the infection data, and the number of *Shigella* used was also the same (MOI = 100 for 0.5 million cells). Signal-to-noise for ^13^C-formate and acetate are shown in Fig. S6 in the supplemental material. Each experiment was performed in triplicate and represented as mean ± SEM. (B) Difference of *Shigella* adherence on HeLa cells at pH 7 with or without 20 mM formate under the conditions marked with the gray box in Fig. 7A.

Second, the metabolic output from *Shigella* was investigated in the adhesion period of the infection model (0 to 1 h) (Fig. 1A). In this period, *Shigella* was mainly extracellular or adhering to the host cells. Due to the dynamics of formate metabolism observed in the growth curve of *Shigella* and the strong pH sensitivity of FocA, two different pH conditions (pH 6 and 7) were used. The same number of *Shigella* was added to an increasingly confluent layer of HeLa cells (0 to >90%) (Fig. 7). A surprisingly strong correlation between the metabolic output from *Shigella* and the number of host cells was observed. At both pH 6 and 7, formate and acetate increased significantly when the confluence of the host cells increased from 0 to 90%. Under the conditions with a fully confluent layer of the host cells, the ratio between acetate and formate was similar for the two pH values (9.2 ± 0.4 and 10.8 ± 1.0, respectively, for pH 6 and 7). *Shigella* produced formate while adhering to the host cells, and adding 20 mM formate during this period did not affect the ability of *Shigella* to adhere to the host cells (Fig. 7B). Thus, other regulators of formate metabolism than the hitherto anticipated strong pH dependence observed for mono-bacterial culture are likely to be in play in an infectious setting.

### Role of formate in host cell defense.

**FIG 8:**
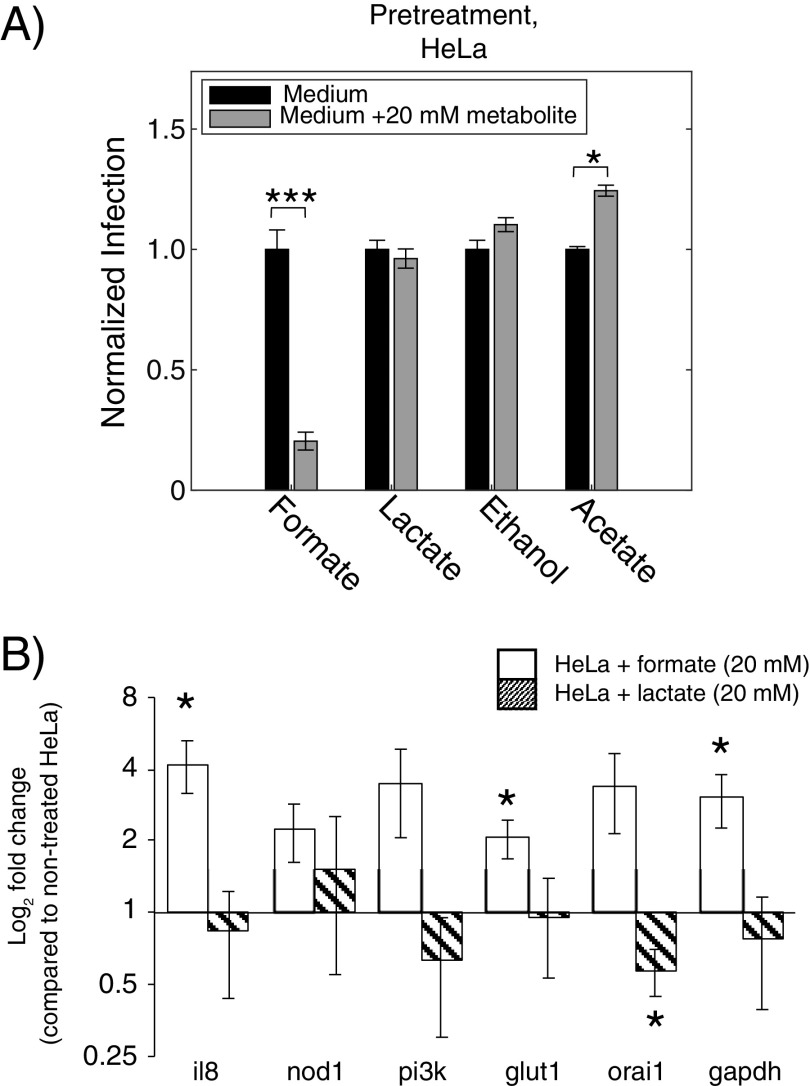
The role of metabolites from bacterial mixed-acid fermentation on HeLa defense. (A) HeLa was treated with 20 mM metabolites formate, lactate, ethanol, and acetate for 3 h before infection (or not). The treatment hereafter continued in the additional 6-h adhesion and proliferation period. Each experiment was performed in triplicate and represented as mean ± SEM. (B) Six selected genes related to immune response (*il8*, *nod1*, *pi3k*, and *orai1*) and glycolysis (*glut1* and *gapdh*) were analyzed with qRT-PCR. The nontreated group relative to 20 mM formate-treated (open bars) group and the nontreated group relative to 20 mM lactate-treated (slash bars) group were analyzed individually. The expression level of each mRNA was calculated according to the ΔΔ*C_T_* method. Data was measured in triplicate and represented as mean ± SEM. Asterisks indicated a statistical difference. *, *P* ≤ 0.05; ***, *P* ≤ 0.001.

**FIG 9:**
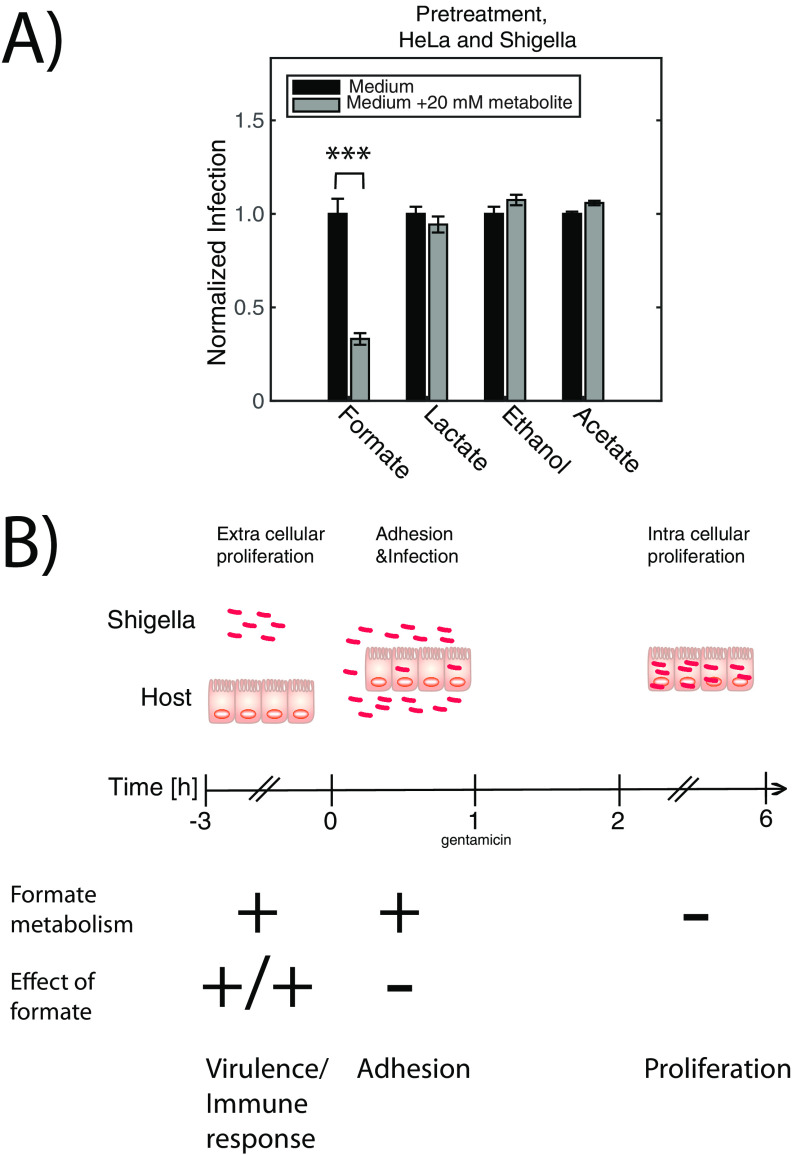
The role of metabolites from bacterial mixed-acid fermentation on *Shigella* and HeLa. (A) HeLa and *Shigella* were treated with 20 mM metabolites acetate, formate, ethanol, and lactate for 3 h before infection (or not). The treatment hereafter continued in the additional 6-h adhesion and proliferation period. Each experiment was measured in triplicate. The results were shown as the mean ± SEM. Asterisks indicated a statistical difference. ***, *P* ≤ 0.001. (B) Summary of the effect of formate in the three different stages of the infection process (separate cell proliferation, adhesion, and intracellular proliferation). Indication of *Shigella’s* ability to produce formate and how both *Shigella* and HeLa cells react to pretreatment with 20 mM formate.

To understand if the host cell defense against pathogens such as *Shigella* is influenced by the presence of formate, we pretreated the host cells ([−3] to 0 h p.i.) with 20 mM of either formate, acetate, ethanol, or lactate and measured the number of internalized *Shigella* (6 h p.i.). Interestingly pretreatment of HeLa with 20 mM formate induced the opposite effect of what was observed with the same treatment of *Shigella*. HeLa cells pretreated with formate showed 80% reduction in the infection rate of *Shigella*, whereas lactate did not show any influence. Acetate slightly increased the infection rate with 10% (Fig. 8A). We then analyzed six inflammation- and metabolism-related genes within HeLa cells with qRT-PCR using 18s rRNA as an internal control (interleukin-8 [*il8*], nucleotide-binding oligomerization domain-containing protein-1 [*nod1*], phosphoinositide 3-kinase [*pi3k*], glucose transporter-1 [*glut1*], calcium release-activated calcium channel protein-1 [*orai1*], and glyceraldehyde 3-phosphate dehydrogenase [*gapdh*]). The genes were selected among major known indicators involved in pathogen infection (Table S2). The gene for the proinflammatory cytokine, *il8*, and the gene for the glucose transporter 1, *glut1*, were significantly upregulated by formate. The glycolytic gene *gapdh* encoding glyceraldehyde-3-phosphate dehydrogenase was significantly upregulated by formate treatment compared to that of the nontreated HeLa cells (Fig. 8B). Upregulation of *gapdh* has previously been shown as response to lipopolysaccharide (LPS) treatment (21). The gene is sometimes used as internal control, but here changes in glycolysis were conceivable and thus 18s rRNA was used (22). Furthermore, for the expression of *nod1* (*P = *0.06), *pi3k* (*P = *0.07), and *orai1* (*P = *0.06), an increasing trend was found after formate treatment. In the lactate-treated HeLa cells, *orai1* was the only gene that was significantly downregulated by lactate compared to the nontreated HeLa cells. Thus, formate increased the immune response in the HeLa cells in addition to increasing the virulence of *Shigella*. In a competing scenario where both *Shigella* and HeLa cells were pretreated with 20 mM formate, the infection rate decreased (Fig. 9A). The decreased infection rate was similar to that obtained when only pretreating the HeLa cells. We did not find any significant change in infection numbers for the three other tested metabolites. In summary, we observed a change in the role that formate played as the infection process progressed (Fig. 9B). When *Shigella* and HeLa cells proliferated separately, *Shigella* produced formate in millimolar amount. The metabolite had a significant impact on the bacterial virulence and also on the host cell immune response. During adhesion, formate was still produced in millimolar concentration, but no effect was observed on *Shigella’s* ability to adhere to the host cell as a function of formate concentration. After internalization of *Shigella*, no accumulation of formate could be detected.

## DISCUSSION

Central carbon metabolism has been put forward as an untapped resource for antimicrobial strategies. Both host cells and bacterial pathogens sense and compete for the same nutrients but with opposite purposes. Metabolism is the first line of defense/attack in infection biology since uptake of energy sources is fast. Metabolites then subsequently regulate the transcriptome and the genome (23). The challenge in targeting central metabolism is understanding the intertwined metabolic networks of the host and the pathogen and how these are regulated (24). Here, we used stable isotope-resolved metabolomics (SIRM) to investigate the dynamics in central carbon metabolism in a cellular infection model. SIRM NMR directly tracks metabolites downstream of the isotope-labeled substrate (25). Arguably, there is a large overlap between host and pathogen metabolic pathways, but even in the core of energy metabolism, the central carbon metabolic pathway, profound differences exist. Facultative bacteria use mixed-acid fermentation to regulate their homeostasis, and the large concentration of these metabolic products are known to influence the health of the host (26). Elevated level of formate in the intestinal lumen has been demonstrated upon intestinal inflammation due to dysbiosis (27). Under anaerobic conditions, bacteria excrete fermentation products such as acetate or lactate, but these products are also formed in the presence of oxygen. In bacteria, this phenomenon is known as overflow metabolism, which corresponds to the Warburg effect known from cancer cells (28). In this study, we found that formate, which is a metabolite from *Shigella* mixed-acid fermentation, has an impact on virulence and immune response that is much larger than that of other small metabolites, lactate, acetate, and ethanol. Formate is produced in millimolar amounts. It is important for the bacterial virulence, but it does not affect adhesion to the host cell. After internalization of *Shigella* into the host cell, formate accumulation is strongly reduced compared to acetate.

Formate metabolism changes during the infection process. How the products of mixed-acid fermentation are regulated depends on oxygen levels since the enzymes that control entry to mixed-acid fermentation, pyruvate formate-lyase, and lactate dehydrogenase (LDH) are negatively controlled by oxygen (29) and pH (30). Upon invasion of *Shigella* into the host cell, we observed a shift in fermentation products where acetate and ethanol were continuously accumulated whereas accumulation of formate was not detected (Fig. 2, 3, and 4). This result is in line with a global proteome analysis which found that the formate transporter FocA was not expressed during intracellular growth (8). In a recent study, it has been shown that increased formate concentration can stimulate the expression of the type III secretion system (T3SS)-related genes such as *ipaJ* and *icsA* in *Shigella*, which facilitated its intracellular spreading (6). It was proposed that *Shigella* senses formate accumulation in the host cytosol to determine bacterial density and regulate virulence factors accordingly. A model was proposed where *Shigella* continuously secreted formate as a by-product. Due to the decreased expression of the formate transporter FocA, loss of FocA would then lead to formate accumulation in the *Shigella* cytosol residing inside the host cell (8). This model was based on *in silico* calculation of *Shigella* metabolism in free culture. Our study suggests that *Shigella* changes its metabolism depending on the cellular environment. Acetate and formate are formed via pyruvate formate-lyase (PFL). Hereafter, formate is further catabolized by enzymes like FDH and in additional pathways such as purine metabolism (31). Whether decreased activity of PFL or increased catabolism is the major driving force for reducing formate concentration remains to be investigated. In *Shigella*, the gene encoding FDH (fdhF), a subunit in formate hydrogenlyase (FHL), has been identified as a potential pseudogene (32). However, it has been demonstrated that FHL in *Shigella* has some activity to catabolize formate even if *fdhF* is a classified pseudogene, but the full mechanism still remains to be investigated (33). A change in formate accumulation alone is difficult to interpret because it can be a result of a general regulation of the full glycolysis or other related pathways. The ratio acetate/formate conversely relates formate to the major catabolic metabolite from *Shigella*, which makes it robust and applicable for comparison between experimental setups across different experimental settings and infection protocols. In free culture under similar conditions to the infection assay (i.e., bacterial number and volume), we find a ratio acetate/formate of 1.5 in Dulbecco's modified Eagle medium (DMEM) and 1.9 in Trypticase soy broth (TSB) (see Fig. S7 in the supplemental material). In the first hour of the infection assay (0 to 1 h p.i.), the ratio acetate/formate was approximately 3. For intracellular *Shigella*, the ratio acetate/formate was determined to >10. In all of the data presented herein, we observe an increased ratio acetate/formate when *Shigella* is in coculture with HeLa compared to when free in solution. We have used different modifications of the gentamicin assay to avoid influence of systematic parameters such as the gentamicin concentration. Potential impact on proliferation of intracellular pathogens due to gentamicin penetration in macrophages has been observed (34), whereas gentamicin was not detected in the cytosol of epithelial Henle cells (35). The assumption is thus that the gentamicin protection assay is a convenient model for bacterial infection in established epithelial cancer cells, such as HeLa, Henle, and Caco-2, but care should be taken upon changing the type of host cells. For HeLa cells, we confirmed that the intracellular production of ^13^C-acetate was not affected by gentamicin concentrations between 30 and 100 μg/mL (see Fig. S8 in the supplemental material). The metabolic data are obtained both on extracellular metabolites, measured directly in the growth medium, which provides minimal manipulation of the cells (Fig. 2 and 3 and Fig. 4A) and intracellular metabolite determination using perchloric acid extraction (Fig. 4B). An increase in intracellular formate concentration from 1.5 to 3 fmol/host cell between noninfected and infected Henle cells has been observed in the literature (6). This measurement is not in full agreement with the metabolic data presented here (Fig. 4), where no increase in formate accumulation was detected. Whether this discrepancy is correlated to different host cells, intracellular multiplicity of infection (MOI), or treatment with bile salts should be evaluated further as more metabolic data on intracellular bacteria become available. Here, we mainly quantified extracellular metabolites, as these measurements require minimal cell manipulation, and we suggest that since the extracellular ratio ^13^C-acetate/^13^C-formate reflects cell homeostasis, it may be used as a convenient probe thereof. Such strategy can be challenged if transport over the mammalian cell membrane is not reflecting the metabolic homeostasis of the infected HeLa cells. However, since cancer cells especially export formate as a by-product due to increased demand of C1 units during high proliferation rates (13), many mammalian cells are capable of active formate transport. Specifically, HeLa cells infected with Staphylococcus aureus excreted formate (36), and formate transport driven by a pH gradient has been observed after internalization of *Salmonella* (12). *Salmonella* and *Shigella* both export small acidic end products from glycolysis in similar amounts (*Salmonella* 10% more than *Shigella*) (see Fig. S9 in the supplemental material). We thus speculate that an intracellular pH gradient is plausible upon *Shigella* infection, which would drive the export of formate similarly to what has been observed for *Salmonella* (12, 37). In our data, we found evidence that formate transport is occurring over the mammalian cell membrane when measuring the amount of unlabeled formate (see Fig. S10B in the supplemental material). The cell medium contains 1 mM pyruvate, which is metabolized to unlabeled formate and acetate. Quantification of these metabolites showed the same pattern as observed for ^13^C-formate and ^13^C-acetate. No significant change was found for either intracellular formate nor acetate, whereas extracellular acetate accumulated in the medium. Interestingly, extracellular formate was significantly decreased in the infected group compared to the cell-free medium, whereas extracellular formate from the noninfected group was increased compared to the cell-free medium (Fig. S10). These data support the discussed literature findings that HeLa cells are capable of transporting formate under the conditions used in the infection assay. They are also in agreement with previous observation that pyruvate is decreased in HeLa cells upon *Shigella* infection (9). Thus, extracellular metabolites seem to play a key role in cellular homeostasis, and the ratio acetate/formate as shown in this work may be an easy and informative way to quantify the bacterial metabolism across the full infection period. For intracellular bacteria, acetate and formate must be actively transported across two cell membranes, first across the bacterial cell membrane and then across the host cell membrane. The extracellular acetate/formate ratio will thus not be a direct proxy for the intracellular concentrations but reflect how both bacteria and host cells produce, catabolize, and transport the two metabolites.

Impact of formate depends on the cell type. It was previously clearly demonstrated that formate increases *Shigella* virulence in Henle cells (6), and we also observe that pretreatment of *Shigella* with 20 mM formate before its infection enhanced the number of the intracellular *Shigella* within HeLa cells (Fig. 5A). Extracellular formate thus influences *Shigella* virulence. Interestingly, pretreatment of HeLa cells with 20 mM formate before *Shigella* infection decreased *Shigella* invasion (Fig. 8A). This showed that the effect of formate was diverse and dependent on measured cell type, HeLa cell, or *Shigella*. Even more important, when pretreating both cell types with 20 mM formate, the *Shigella* infection rate was decreased to the same level as when only HeLa cells were pretreated with formate (Fig. 9A). Formate thus induces a beneficial response in the host cell, which can outcompete the increased virulence of *Shigella*. Previously, it has been shown that treatment of Caco-2 cells with 5 mM formate could restore a drug-induced decrease in transepithelial electrical resistance (TEER) (38). TEER is one of the most important measures of tight junctions, which prevent penetration of pathogens. The kinase PI3K has also been connected to tight junctions. PI3K activates the expression of the downstream gene *cldn2*, encoding the tight junction protein in Caco-2 cells (39). Although we did not observe a significant difference for PI3K between nontreated and formate-treated HeLa cells (*P = *0.078), our findings indicate a positive trend toward PI3K upregulation. In addition, activation of the PI3K/AKT pathway has been demonstrated to enhance glucose uptake (40). We found a significant upregulation of two genes from the glycolysis *glut1* and *gapdh* supporting the trend of PI3K. The general marker for immune response interleukin-8 (IL-8) was significantly upregulated in our study. It has previously been shown that activation of PI3K/AKT also involved upregulation of IL-8 production (41). Thus, similar regulatory network of the *il8* activation via the PI3K pathway was found here. The upregulated IL-8 also indicates that the nuclear factor-κB (NF-κB) pathway, one of the major pathways of host immune responses, is activated. NF-κB regulates subsequent pathways such as NADPH oxidase (NOX), which clears the invaded pathogens via the production of reactive oxygen species (ROS) (42, 43).

Formate increased *Shigella* virulence. In *Shigella*, our analysis revealed that T3SS-related genes including *virF*, *ipgD*, and *icsA* were significantly upregulated by formate treatment (Fig. 5B). This finding supports suggestions in the literature that formate could be an enhancer of *Shigella* invasion (6). In a previous study, additional formate was shown to affect the flux through PFL, which reversed and regenerated pyruvate (44). In our data, the expression of *pflB* and *pta* was significantly upregulated when *Shigella* was treated with formate. This could point to mixed-acid fermentation actively reusing formate when it is present in high concentration to generate the high-energy metabolite pyruvate. A past study showed that adding formate to E. coli downregulated the expression of 6-phosphofructokinase *pfkB* (45). How mixed-acid fermentation and the upper part of glycolysis work together under high formate concentration is still elusive.

Formate did not affect adhesion. Treatment of *Shigella* with formate enhanced its pathogenicity both in this and in previous studies (6). However, the accumulation of the endogenous formate within *Shigella* was decreased after host cell invasion. Our result also indicated that pretreatment with formate on HeLa cells could increase the host defense and thereby diminish the intracellular proliferation of *Shigella*. Together these evidences imply that the effect of formate was diverse, and the effect depended on the timing of the treatment. We generated a growth curve of *Shigella* and determined the pH value of medium at different time points together with an analysis of the composition of metabolites. The growth curve showed that the log-phase of *Shigella* started 2 h after incubation (Fig. 6), and the initial pH value was around 7.5. After 3 h incubation, *Shigella* reached the mid-log stage, and the pH had reduced to 6.0 and it kept decreasing until 5.5 at 5 h. Meanwhile, the metabolic analysis showed that the consumption of glucose and production of acetate and ethanol followed inversely proportional and matched the result from the *Shigella*-infected HeLa, where we also observed a continuous production of acetate and ethanol. Interestingly, formate was sharply increased during the first 2 h, but it was dramatically decreased after 4 h. Previously, the expression level of one of the major virulent proteins, IcsA, within *Shigella* has been found to have a maximum at the mid-log stage during *Shigella* growth in rich medium, and then it was reduced (46). In addition, the type three secretion apparatus (T3SA) within T3SS in *Shigella* is upregulated during the initial bacterial entry and downregulated when *Shigella* successfully enters into the host cytosol (47). Our observations imply that formate accumulation follows the same pattern as T3SS, namely, that it is increased upon infection and then reduced once the bacteria are internalized. This would also be in accordance with similar studies of E. coli, where it was observed that once pH drops below 6.8, formate is completely imported back into the cells (48). This characteristic switch in formate metabolism has been connected to the transporter FocA, and a thorough mechanistic study of this protein confirmed a remarkable pH switch for FocA (49). The enzyme switched from a passive channel to an active transporter at pH 5.1 to 5.6. There is, thus, a discrepancy in the understanding of formate metabolism. In bacterial cells, the switch occurs at pH 6.8, whereas in the enzymatic studies of FocA, the switch occurs below pH 5.6. FocA belongs to a family of transporters (FNT) and it has been shown that the NirC transporter, another member of the FNT family, has half the transport rate of formate compared to FocA (50). Likewise, FocA has been shown to transport acetate with relatively high affinity (49). This implies that the regulation of formate metabolism is more complex than hitherto suggested. We, thus, compared the formate accumulation as a function of host cell confluence at pH 6 and 7. In contrast to what was observed in free culture, formate accumulation in *Shigella* was not abolished at pH below 6.8. On the contrary, it was highly stimulated by interaction with the host cell both at pH 6 and 7. This increased formate metabolism in proximity of the host cell align well with observations by us and others that formate increases *Shigella* virulence, and it suggests that the controlling factor for formate metabolism cannot exclusively be attributed to a pH effect. Other effectors must be at play that have not yet been identified. Reactive oxygen species (ROS) might be such effectors. Increased secretion of ROS, one of the important innate immune responses, was induced by lipopolysaccharides (LPS) from *Shigella* in HeLa cells (51). It was demonstrated that LPS controlled *Shigella* T3SS expression and its viability (52, 53). Currently, it is not clear if there is a correlation between ROS and formate metabolism during *Shigella* infection. However, in *Salmonella*, the expression of pyruvate formate-lyase (*pflB*) was downregulated upon treatment with 1 mM H_2_O_2_ (54). Under this oxidative pressure, Salmonella showed the ability to continue acetate and lactate production (55). Importantly ROS production in host cells induced by *Shigella* occurred 5 h p.i. (56), which is later than the change in formate metabolism observed in our study (1 h p.i.). Whether ROS and formate metabolism are correlated, thus, still needs to be clarified.

In conclusion, we demonstrate the importance of a detailed understanding of the regulation of pathogen metabolism. We show that formate not only regulates bacterial virulence but also the immune response of the host cell. We also demonstrate that *Shigella* metabolism is different between free culture, upon adhesion to the host cells, and once it has entered the host cell.

## MATERIALS AND METHODS

### Bacterial strain and growth condition.

The Shigella flexneri serotype 2a ATCC 700930 (alternatively annotated as 2457R) was purchased from American Type Culture Collection (ATCC) (Manassas, VA). The bacteria were recovered from −80°C stock by streaking onto 0.01% Congo red (Sigma-Aldrich, St. Louis, MO) agar and incubated at 37°C overnight. The processes of the routine bacterial growth and the culture for doing the infection assay are described in the supplemental material.

### HeLa cell culture and gentamicin protection assay.

The HeLa CCL-2 (human cervical carcinoma) cells obtained from ATCC were generally maintained in the Dulbecco's modified Eagle medium (DMEM) (Gibco, Carslbad, CA) containing 4.5 g/L d-glucose, 4 mM l-glutamine, 1 mM sodium pyruvate, and supplemented with 10% fetal bovine serum (FBS) (Sigma) under 5% CO_2_ atmosphere at 37°C, and the cells were passaged every 3 to 4 days by diluting a suspension of the cells in fresh medium. The procedures for cell seeding and gentamicin protection assay are explained in detail in Fig. S1 and Fig. S11 in the supplemental material. For metabolic investigations, DMEM medium was replaced with minimal DMEM containing 20 mM [U-^13^C] glucose as the carbon source.

### ^1^H NMR analysis.

All medium samples were stored at −18°C until NMR analysis. Samples were thawed, and 300 μL of the samples was prepared by adding 250 μL of 40 mM deuterated phosphate buffer with DSS (3-(Trimethylsilyl)-1-propanesulfonic acid sodium salt) as a standard (40 mM, 0.2 mM DSS, 100% D_2_O, pH 7.5). One-dimensional (1D) ^1^H spectra were recorded using a standard Bruker pulse sequence noesygpr1d (TD 32768; interscan delay (d1), 4 s; acquisition time, 1.7 s; number of scans, 128). In total, each spectrum took approximately 12 min to acquire. All spectra were acquired at 298 K on an 800 MHz Avance III NMR spectrometer (Bruker, Switzerland) with a TCI z gradient CryoProbe. Subsequently, the data was analyzed using the MNova software.

For absolute quantification of formate and glucose, two individual standard curves were prepared each with 5 concentrations in the range of 0 to 20 mM in the same media as used for *Shigella* growth.

### Metabolite treatment of Shigella and HeLa cells.

For determining the effect of the metabolites produced from glycolysis on *Shigella* during the infectious process, a 1:100 dilution of the overnight cultured *Shigella* was subinoculated in fresh TSB supplemented with 20 mM each metabolite and grown for 3 h until reaching the mid-log stage. In addition, for determining the effect of each metabolite on HeLa cells, the HeLa cells seeded on a 12-well plate were pretreated with DMEM supplemented with 20 mM of each metabolite for 3 h before *Shigella* infection. The detailed procedures are described in the supplemental material.

### Determination of the mRNA expression in Shigella and HeLa cells.

The total RNA samples from HeLa cells and *Shigella* cells treated with 20 mM sodium formate and sodium lactate and the nontreated cells of both HeLa and *Shigella* were isolated and purified. The expression level of each mRNA was determined by the quantitative real-time reverse transcriptase-PCR (qRT-PCR) analysis. The procedure is described in Tables S1 and S2 in the supplemental material.

### Statistical analysis.

The statistical analyses were calculated using analysis of variance (ANOVA). A *P* value of ≤0.05 was considered a significant difference.
