# Supplementary material for: Formate Metabolism in Shigella flexneri and Its Effect on HeLa Cells at Different Stages during the Infectious Process
Source: Microbiol Spectr. 2023 Apr 12;11(3):e00631-22. doi: 10.1128/spectrum.00631-22 (PMC10269805; doi:10.1128/spectrum.00631-22)
Supplement: Supplemental file 1 — Supplemental material. Download spectrum.00631-22-s0001.pdf, PDF file, 2.5 MB [file spectrum.00631-22-s0001.pdf]

SUPPLEMENTARY INFORMATION

Formate metabolism in *Shigella flexneri* and its effect on HeLa cells at different stages during the infectious process

Ke-Chuan Wang[1], Mathilde Hauge Lerche[1], Jan Henrik Ardenkjær-Larsen[1], Pernille Rose Jensen[1*]

[1]Dept. of Health Technology, Technical University of Denmark, Kgs. Lyngby, Denmark

To whom correspondence should be addressed:
Pernille R. Jensen,
Department of Health Technology,
Technical University of Denmark,
Oersteds Pl. Bldg. 349, Room 120, 2800 Kgs. Lyngby.
Telephone: (+45)45253688;
E-mail: peroje@dtu.dk.

Table of content

Figure S1. *Detailed infection procedures for sampling metabolites.*
Figure S2. *Analysis of full ^{1}H spectrum for metabolites responsible for discrimination between infected and non-infected HeLa cells.*
Figure S3. *Representative ^{1}H NMR spectra of intracellular metabolites from PCA extracted cells at 6-h PI for HeLa cells and infected HeLa cells (from Fig. 4A).*
Figure S4. *Standard curves for ^{1}H NMR quantification of ^{13}C formate and ^{13}C acetate.*
Figure S5. *dDNP-NMR analysis of total ^{13}C metabolites produced from HeLa cells and Shigella-infected HeLa cells.*
Figure S6 *Representative ^{1}H NMR spectra of extracellular metabolites from Shigella metabolism in presence of HeLa cells (from Fig. 7A).*
Figure S7. *Extracellular metabolites from Shigella grown in DMEM and TSB.*
Figure S8. *Effect of treating different concentrations of gentamicin on Shigella infection.*
Figure S9. *Extracellular metabolites from Shigella and Salmonella in TSB.*
Figure S10. *^{1}H NMR analysis of unlabelled metabolites produced from HeLa cells and Shigella-infected HeLa cells.*
Figure S11. *Optimization of Shigella infection in HeLa cells*
Materials
Table S1. *Primers used for qRT-PCR*
Table S2. *Detailed information on selected genes involved in pathogen infection*

Supplemental Information

Figure S1.

Detailed infection procedures for sampling metabolites.

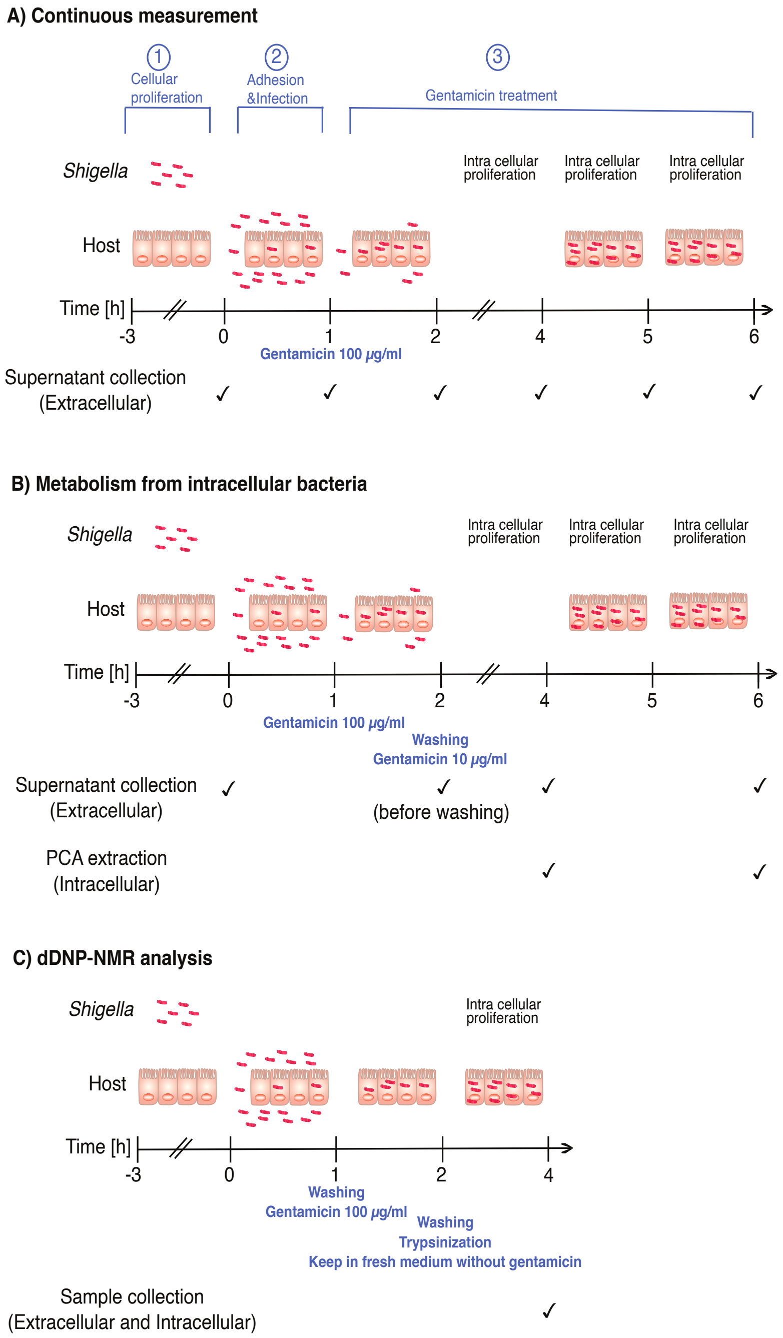

Figure S1. Detailed infection procedures used to sample metabolites from Shigella-infected HeLa cells for NMR analysis. *(A) Continuous analysis of metabolites from the supernatant*

of the Shigella-infected HeLa cells. Gentamicin (100 µg/ml) was directly added into each well 1-h (1-h PI) after Shigella infection and the concentration was kept for additionally 5-h (6-h PI) without medium replacement. Samples for NMR analysis were collected every hour. (B) For analyzing the metabolites from the intracellular Shigella a comparison was performed between the metabolic conversion in the initial infection period (0-2-h) and the intracellular proliferation period (4-6-h). The standard gentamicin protection assay was performed where 100 µg/ml of gentamicin was added after the first 1-h (1-h PI). The sample was incubated for additional 1-h (2-h PI). At 2-h PI, the supernatant was collected, and then the well was washed by PBS twice and the medium was also replaced with fresh medium containing 10 µg/ml of gentamicin. Each well was kept incubating for additional 4-h. The supernatants from each well were collected at 0-h PI, 2-h PI, 4-h PI, and 6-h PI. For determination of intracellular metabolites the cells were washed with PBS and the extracted with ice cold perchloric acid. (C) For running dDNP-NMR, the gentamicin treatment (100 µg/ml) was performed at 1-h PI and the sample was incubated for additional 1-h (2-h PI). Then, the Shigella-infected HeLa cells were trypsinized and incubated in fresh medium without gentamicin for 2-h (4-h PI). The total metabolite content (intracellular plus extracellular) was recovered by PCA extraction. Additional details about the infection protocols can be found under SI, Material section, and Fig. S10.

Figure S2.

Analysis of full ^{1}H spectrum for metabolites responsible for discrimination between infected and non-infected HeLa cells in the continuous assay.

NMR spectra recorded 6-h PI following the procedure of the continuous measurement of metabolism were divided into buckets of 0.02 ppm. A supervised principal component analysis (PLS-DA) clearly separated the two groups, infected and non-infected (Fig. S1A) and the biplot from a non-supervised PCA analysis finds strong correlation between ^{13}C-aceate, ^{13}C-ethanol, ^{13}C-formate and unlabeled acetate (Fig. S1B).

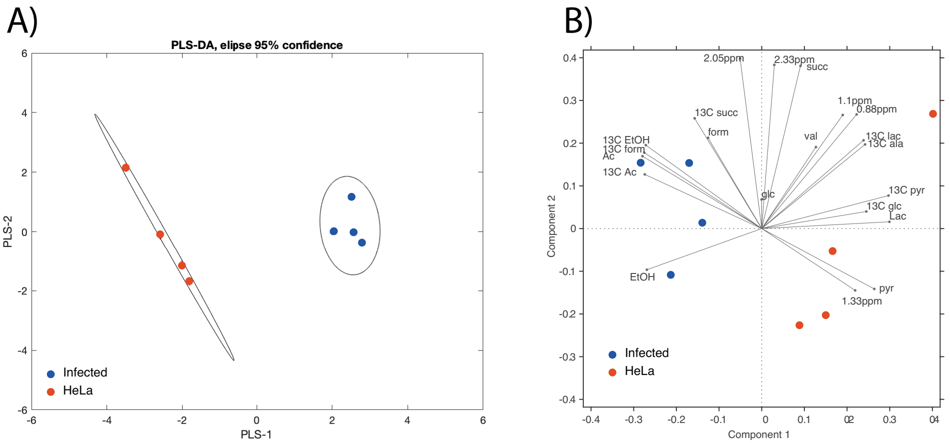

***Figure S2**. Principal component analysis A) Supervised principal component analysis (PLS-DA) clearly separated the two groups, infected and non-infected HeLa cells (n=4 for each). Biplot from unsupervised PCA analysis identified correlation between ^{13}C acetate, ^{13}C ethanol, ^{13}C formate and acetate.*

Figure S3.

Representative ^{1}H NMR spectra of intracellular metabolites from PCA extracted cells at 6-h PI for HeLa cells and infected HeLa cells (from Fig. 4A).

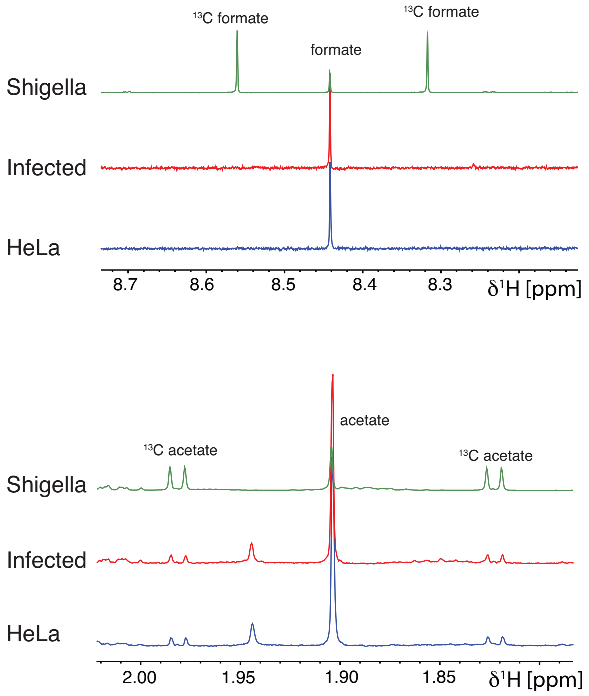

Figure S3. Representative ^{1}H NMR spectra of intracellular metabolites from PCA extracted cells at 6-h PI for HeLa cells and infected HeLa cells (from Fig. 4A). *To show the position of the missing ^{13}C formate peaks a spectrum from Shigella is included.*

Figure S4.

Standard curves for ^{1}H NMR quantification of ^{13}C formate and ^{13}C acetate.

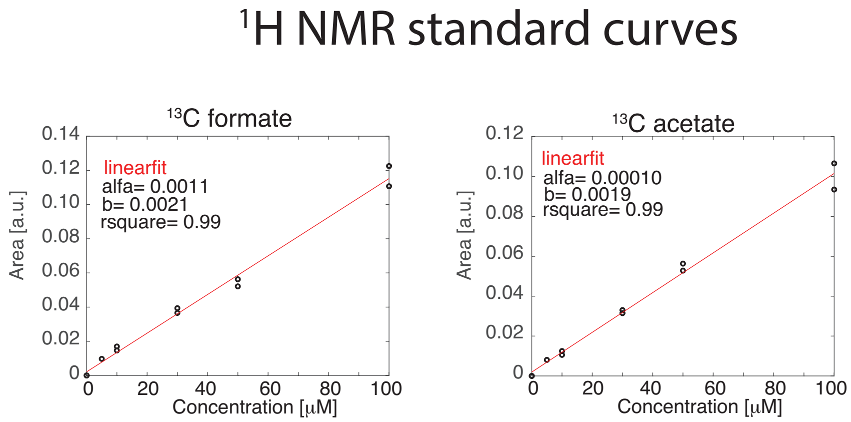

Figure S4. ***^{1}H NMR quantification of ^{13}C formate and ^{13}C acetate****. The detection limit and standard curve was performed for ^{13}C formate and ^{13}C acetate in DMEM medium. Two individual stock solutions of 0.1 M ^{13}C formate and ^{13}C acetate in water were serial diluted to the concentration range 5 μM to 100 μM in DMEM medium. ^{1}H NMR spectra were recorded with identical parameters as used for the biological samples. A linear fit was used to determine the response factor (the slope of the fit) for each metabolite.*

Figure S5.

dDNP-NMR analysis of total ^{13}C metabolites produced from HeLa cells and Shigella-infected HeLa cells.

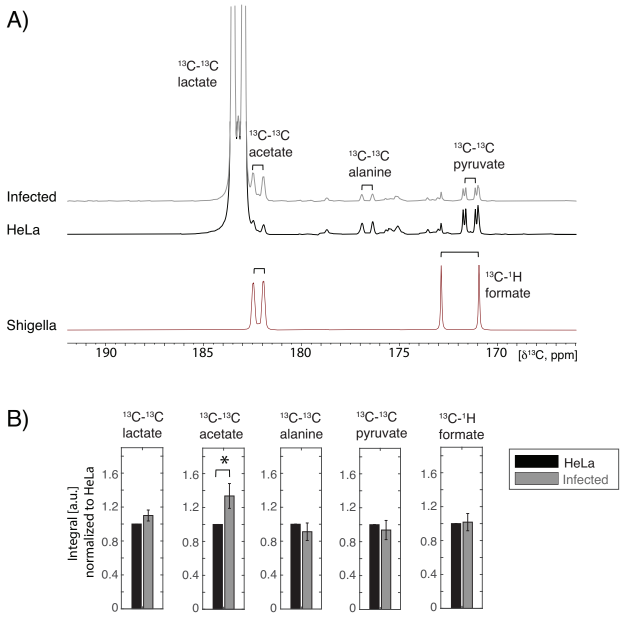

***Figure S5. dDNP-NMR analysis of total ^{13}C metabolites produced from Shigella alone, HeLa cells, and Shigella-infected HeLa cells**. [U- ^{13}C] labelled glucose was used as substrate during 2-4 h PI. (A) The ^{13}C spectrum from dDNP-NMR of metabolite extracts from Shigella-infected HeLa cells, HeLa cells and Shigella. (B) Levels of ^{13}C lactate, ^{13}C acetate, ^{13}C alanine, ^{13}C pyruvate and ^{13}C formate obtained in infected HeLa cells compared to the levels in non-infected HeLa cells. Data are represented as mean ± SEM and asterisk indicated a statistical difference ($p \leq 0.05$), n=8.*

Figure S6.

Representative ^{1}H NMR spectra of extracellular metabolites from Shigella metabolism in presence of HeLa cells (from Fig. 7A).

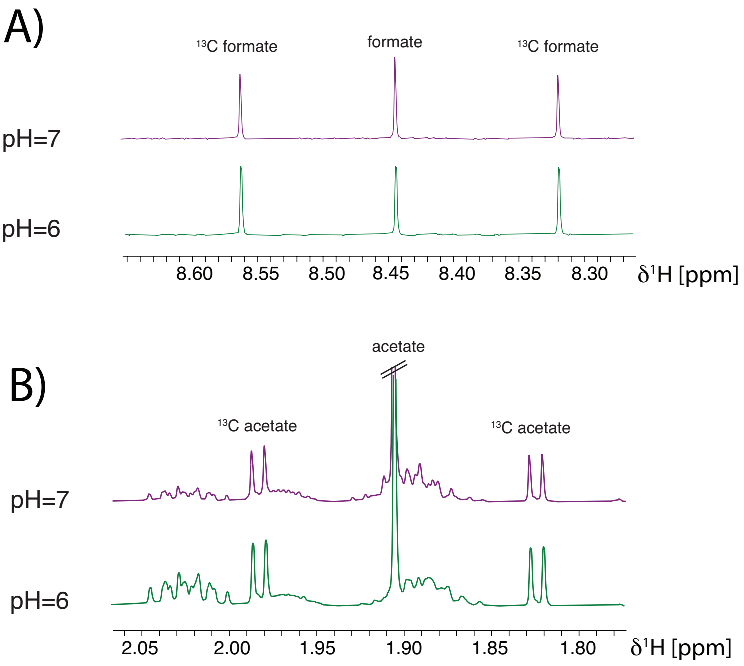

Figure S6. Representative ^{1}H NMR spectra of extracellular metabolites from Shigella metabolism in presence of HeLa cells (from Fig. 7A). *A) Region showing ^{13}C formate signals from 90% confluent HeLa cells at pH 6 and 7. B) Region showing ^{13}C acetate signals from 90% confluent HeLa cells at pH 6 and 7.*

Figure S7.

Extracellular metabolites from Shigella grown in DMEM and TSB.

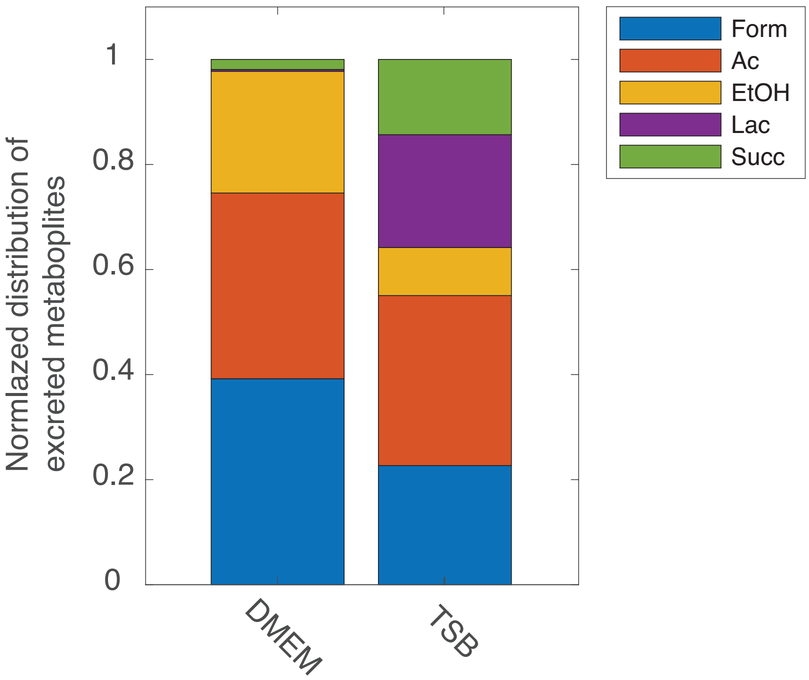

***Figure S7. Extracellular metabolites from Shigella grown in DMEM and TSB**. Samples from the growth medium were taken right before inoculation and after 6 hours of growth. ^{1}H NMR was acquired according to protocol. Integrals from the different metabolites were divided with the respective number of protons in functional groups and the background (t=0 h) was subtracted.*

Figure S8.

Effect of treating different concentrations of gentamicin on Shigella infection.

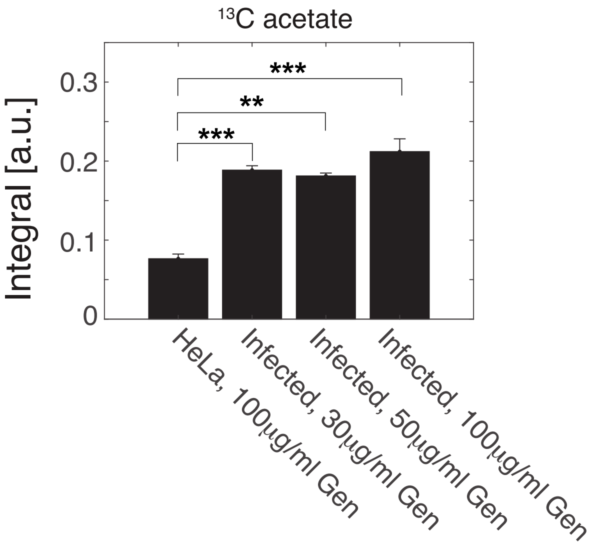

Figure S8. Effect of treating different concentrations of gentamicin on Shigella infection. *The ^{13}C acetate production from Shigella-infected HeLa cells was analyzed by ^{1}H NMR 6-h PI following the gentamicin protection assay for measuring metabolism from intracellular bacteria (Fig. S1B). Briefly, 1:100 MOI of mid-log cultured Shigella was infected in HeLa cells and was incubated for 1-h at 37oC. Then, each medium was replaced by the gentamicin-containing media with 30 µg/ml, 50 µg/ml, and 100 µg/ml of gentamicin separately. Meantime, the group of non-infected HeLa cells was treated with 100 µg/ml of gentamicin as the control. After additional 1-h incubation, all the media were replaced again by the media containing low-concentration of gentamicin at 10 µg/ml for an additional 4-h incubation. N=2, statistical analysis was performed by ANOVA and significant differences were shown as $^{**}p \leq 0.01$ and $^{***}p \leq 0.001$.*

Figure S9.

Extracellular metabolites from Shigella and Salmonella grown in TSB.

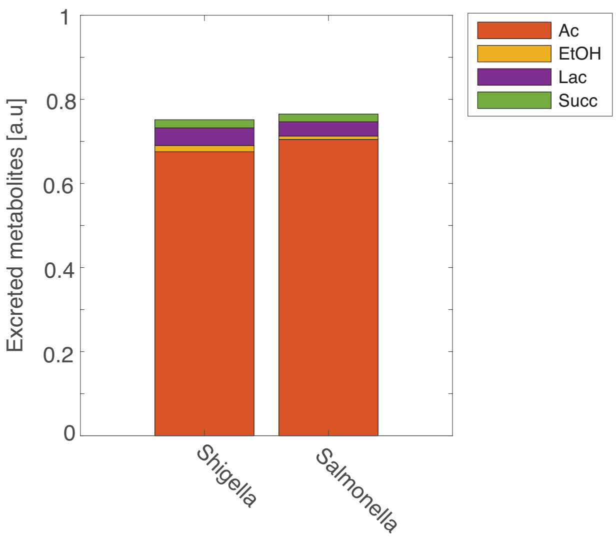

Figure S9. Extracellular metabolites from Shigella and Salmonella. *Both bacteria were grown in TSB medium at 37°C with shaking at 200 rpm to OD_{600} = 0.8. ^{1}H NMR was acquired according to protocol. Integrals from the different metabolites were divided with the respective number of protons in functional groups and the metabolites from pure medium was subtracted. Four of the main products from the mixed-acid-fermentation was produced with acetate contributing with more than 90% of the total metabolic output for both Shigella and Salmonella.*

Figure S10.

^{1}H NMR analysis of unlabelled metabolites produced from HeLa cells and Shigella-infected HeLa cells.

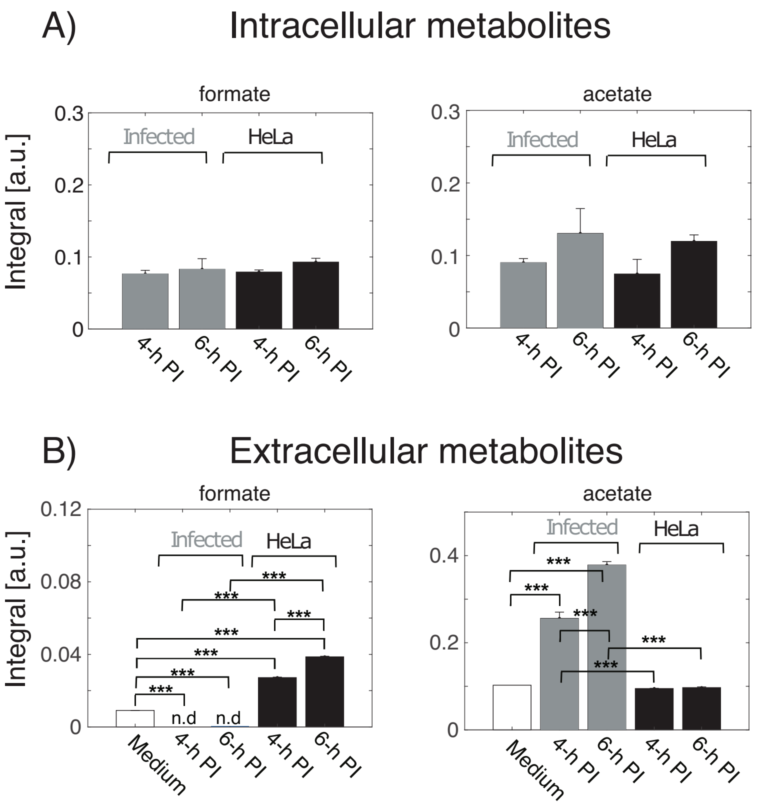

Figure S10. ***^{1}H NMR analysis of unlabelled metabolites produced from HeLa cells and Shigella-infected HeLa cells****. A) Intracellular metabolites from PCA extracted cell samples at 4-h and 6-h PI showed no production of intracellular unlabelled formate whereas a non-significant increasing tendency was observed for acetate in both groups. Unlabelled metabolites were derived from intrinsic pyruvate within the minimal DMEM medium. B) The supernatants collected at 4-h and 6-h PI showed that excreted unlabeled formate significantly decreased relative to medium without cells for both time points in the infected group whereas formate significantly increased in supernatants from HeLa cells. For acetate a significant increase was observed for both time point in the infected group whereas no significant production could be detected in the supernatants from HeLa cells. Asterisk indicated the statistical difference ($p \leq 0.05$) by ANOVA. Data are represented as mean ± SEM (n=3).*

Figure S11.

Optimization of Shigella infection in HeLa cells

Shigella is a typical intracellular pathogen which can actively induce the membrane ruffling of host cell and be internalized inside cytosol. A well described cellular infection model based on the gentamicin protection assay was applied using HeLa cells as host. In the initial infection period, which was varied between 30 and 120 min, a 100-fold excess of *Shigella* (MOI=100) was used. The infection numbers were not critically depending on the infection period and hence forward an infection period between 45 and 60-min was used based on this. After the infection period the antimicrobial drug gentamicin was added and the intra cellular bacterial number was hereafter followed over time. The result showed that the highest number of invaded *Shigella* was 7.9 after 5-h proliferation, and the invasion rate was decrease at 6-h. The cell viability of the HeLa cells after *Shigella* infection under the applied conditions was stable as determined by the MTT assay and were therefore considered functionally uncompromised.

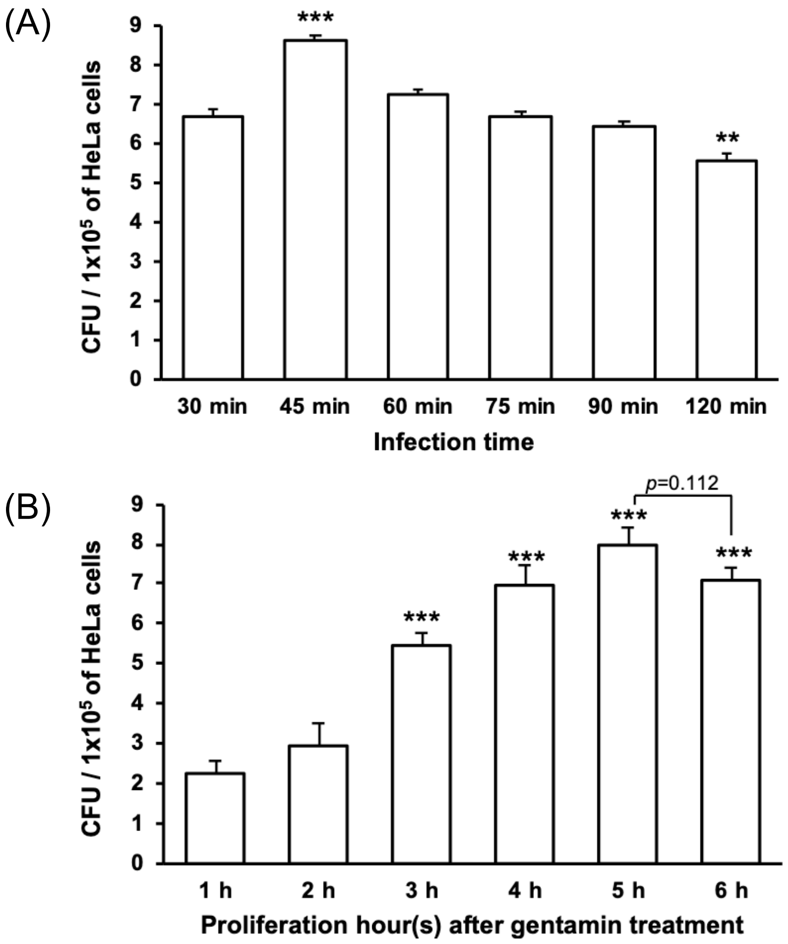

Figure S11. Optimization of the Shigella infection condition for gaining the optimal number of invaded Shigella using the HeLa cell-based model. *(A) To determine the optimal infection time before gentamicin treatment the infection protocol in Fig. S1B was followed. 1 × 10^5 of HeLa cells were infected with Shigella at 100 of MOI for 30-120-min and the cells continuously proliferated for 6-h after replacement of the gentamicin medium. Then, the number of the intracellular Shigella was counted on agar plates. (B) To optimize the proliferation time after gentamicin treatment, 1 × 10^5 of HeLa cells were infected with Shigella at 100 of MOI for 60-min and were continuously proliferated for 1-6-h after replacement of the gentamicin medium. Then, the number of the intracellular Shigella was counted on agar plates as well. Each experiment was performed in triplicate and the results were presented as the mean ± SD. The p-values were obtained by Student's t-test, * is $p \leq 0.05$, ** is $p < 0.01$, and *** is $p \leq 0.001$.*

Materials

Bacterial growth and maintenance for the infection assay

For each infection assay, one single colony from the agar plate was inoculated into tryptic soy broth (TSB) medium (Sigma) and was incubated at 37°C with shaking at 200 rpm overnight. Then, 1:100 dilution from the overnight culture was sub-cultured into fresh TSB and was incubated again at 37°C with shaking at 200 rpm for 3-h to reach mid-log stage at $OD_{600} = 1$. To enhance *Shigella* adherence to host cells, the mid-log cultured bacteria were washed by PBS twice and resuspended in 0.001% poly-L-lysine solution (Merck Millipore, Billerica, MA), and then incubated at 37°C with shaking at 100 rpm for 15-min. Hereafter, the bacteria were washed by PBS again and resuspended in serum-free medium. The current number of bacteria was determined by measuring OD_{600}. Then the correct number of bacteria needed for multiplicity of infection 100:1 (MOI 100) was calculated based on a previously determined conversion number of *Shigella* that $OD_{600} = 1$ approximately equaled 8×10^8 cells/ml of *Shigella*.

The gentamicin protection assay and medium collection for NMR analysis

Minimal DMEM was always supplemented with 25 mM HEPES, 1 mM sodium pyruvate and 4 mM glutamine.

1. Continuous measurement of metabolism

5×10^5 HeLa cells per well were seeded onto 12-well plates (VWR International, Batavia, IL) in DMEM medium and grown at 37°C with 5% CO_2 overnight. On the experiment day, the culture medium was exchanged to the serum-free DMEM for 3-h. At the same time, the overnight culture of *S. flexneri* was sub-cultured to mid-log phase as the above

description. Then, the HeLa cells were infected with 100:1 multiplicity of infection (MOI) of the mid-log *S. flexneri* that was re-suspended in serum-free minimal DMEM with 20 mM [U-^{13}C]-glucose. The infected HeLa cells were incubated at 37 °C with 5% CO_2. At 1-h PI 100 µg/ml gentamicin was added into the growth medium of the infected HeLa cells and samples for continuous metabolism were collected each hour until 6-h PI. Hereafter, the number of intracellular *Shigella* was determined by plating the lysed cells onto Congo red agar. The group of non-infected HeLa cells was performed as above omitting the *Shigella* infection part.

2. Determination of metabolism from intracellular bacteria

After the initial infection performed as above, the medium of each well was replaced with serum-free minimal DMEM supplemented with 20 mM [U-^{13}C]-glucose. After 1-h (1-h PI) gentamicin 100 µg/ml was added and the medium was collected for NMR analysis 2-h PI. Then, the cells were washed with PBS and new minimal DMEM medium was added supplemented with 10 µg/ml gentamicin (Sigma) and 20 mM [U-^{13}C]-glucose for additional 4-h incubation. The supernatants from both non-infected HeLa and infected HeLa cells at 0-h, 2-h, 4-h, and 6-h PI were stored for directly analyzing extracellular metabolites. After removing the supernatant, both non-infected HeLa and infected HeLa cells at 4-h and 6-h PI were washed and directly lysed by adding ice-cold 2.2 M perchloric acid (PCA) solution for the following PCA extraction according on a general protocol (1). Briefly, each lysed cell sample was centrifuged for removing the cell debris, and the pH was adjusted to neutral. Then, the neutralized sample was freeze-dried and was used for the subsequent ^{1}H NMR analysis.

3. For the dDNP-NMR analysis the infection protocol was scaled up

1×10^7 HeLa cells were seeded onto a T-75 flask and were cultured at 37°C with 5% CO_2 overnight before the infection day. The normal infection procedure was hereafter followed. 1-h PI the medium was replaced by serum-free minimal DMEM containing 100 µg/ml gentamicin and was incubated for an additional 1-h (2-h PI). Hereafter, two flasks of the *Shigella*-infected HeLa cells were trypsinized and combined into one Eppendorf tube with the replacement of fresh-prepared minimal DMEM containing 20 mM [U-^{13}C] glucose. The cells were incubated for 2-h and the metabolites were extracted by PCA extraction procedure and used for dDNP-NMR analysis. For doing ^{13}C dDNP NMR analysis, The lyophilized extracts of metabolites was analyzed with dDNP-NMR according to previously described protocol (1). In brief the metabolites are mixed with a glassing matrix containing a stable radical. The sample was polarized for 90 min using a Hypersense polarizer and subsequently dissolved with 5 ml hot buffer. A 1D ^{13}C spectrum was acquired with a 90° pulse on a 9.4 T Varian spectrometer 8 s after dissolution.

Metabolites treatment on Shigella and HeLa cells

For determining the effect of metabolites produced after glycolysis on *Shigella* during the infectious process, the 1:100 dilution of the overnight cultured *S. flexneri* was sub-inoculated in fresh TSB supplemented with 20 mM of each metabolite including sodium acetate (Sigma), sodium formate (Sigma), sodium lactate (Sigma), and ethanol (VWR BDH Chemicals, Westchester, PA) independently. Four groups were compared to investigate the effect of the metabolites on *Shigella* and HeLa cells separately:

(1) Normal infection procedure as described above.

(2) Pre-treatment of *Shigella* cells:

Shigella was grown for 3-h at 37°C with shaking at 200 rpm to reach mid-log phase in presence of either 20 mM formate, acetate, lactate or ethanol. After 3-h each metabolite pre-treated or non-treated *Shigella* was infected into HeLa cells which were already seeded in 12-well plate overnight following the procedure of gentamicin protection assay and was incubated at 37°C for 60 min. Then, the medium was removed and replaced by serum-free DMEM including 100 µg/ml gentamicin and 20 mM of each metabolite for an additional 1-h of incubation. After treatment with high concentration of gentamicin, each medium was switch to serum-free DMEM including 10 µg/ml gentamicin and 20 mM of each metabolite for an additional 4-h of incubation. At the final time point, each group of infected HeLa cells were lysed by 1% Triton X-100 solution and plated onto Congo red agar plate for counting the number of intracellular *Shigella*.

(3) Pre-treatment of HeLa cells:

Overnight *Shigella* was sub-cultured in normal TSB for 3-h at 37°C with shaking at 200 rpm to mid-log. At the same time, the medium of HeLa cells which were already prepared in the 12-well plate following the above description was exchanged with serum-free DMEM supplemented with 20 mM of each metabolite for 3-h (-3-0-h PI). The above procedure of gentamicin protection assay was hereafter followed i.e each of the four metabolites was kept at 20 mM during the rest of the infection procedure.

(4) pre-treatment in both HeLa cells and *Shigella*

Shigella was grown for 3-h at 37°C with shaking at 200 rpm to reach mid-log phase in presence of either 20 mM formate, acetate, lactate or ethanol. At the same time, the medium of HeLa cells which were already prepared in the 12-well plate following the above

description was exchanged with serum-free DMEM supplemented with 20 mM of each metabolite for 3-h (-3-0-h PI). The above procedure of gentamicin protection assay was hereafter followed i.e each of the four metabolites was kept at 20 mM during the rest of the infection procedure.

Quantitative real-time reverse transcriptase-polymerase chain reaction (qRT-PCR)

The total RNA samples from HeLa cells and *S. flexneri* cells treated with 20 mM sodium formate and sodium lactate, and the non-treated cells of both HeLa and *S. flexneri* were isolated and purified by the GeneJET RNA purification kit (Thermo Fisher Scientific, Ottawa, Ontario) followed the manual instruction. After RNA purification, one microgram of each RNA sample was treated with one unit of DNase I (New. England BioLabs, Beverly, MA) at 37°C for 10-min for removing the residual genomic DNA and at 75°C for another 10-min incubation for inactivating the enzyme digestion. Then, each purified RNA was reverse-transcribed to the complementary DNA (cDNA) performed by the iScript cDNA synthesis kit (Bio-Rad, Hercules, CA). According to the manual guidance, the oligo-dT primer and random hexamer primer were used for generating the cDNA from the purified RNA of HeLa and *S. flexneri* separately. Then, each mixture was incubated at 25°C for 5-min and at 42°C for 30-min for cDNA synthesis and was heated at 85°C for 5-min to stop the reaction. After cDNA preparation, the qRT-PCR was performed in triplicate by using the iQ SyBr green super mix kit (Bio-Rad). Briefly, 50 ng of each cDNA was reacted with the specific primers (Table S1 and Table S2) on the CFX96 real time PCR system (Bio-Rad) using the following program: 95°C for 3 min; followed by 40 cycles of 95°C for 15-sec, 46°C for 30-sec and 72°C for 30-sec for DNA amplification. The housekeeping genes of the *18s* and *16s* ribosomal RNA within the HeLa and *S. flexneri* cells individually were used as the internal. The expression levels of these genes were calculated based on the $^{\Delta\Delta}$Ct method (2). Each value

from the formate and the lactate-treated group was shown as the relative expression level and was compared to the non-treated group. All data were presented as geometric mean ± standard error of the mean (SEM).

Table S1. Primers used for qRT-PCR

- For *Shigella*

Gene name	Sequence (5'→3')	Function	Tm
virF	*virF*-F: 5'- TTAGCTCAGGCAATGAAACT -3'<br>*virF*-R: 5'- TTAACCCCCTTTTCTCCTCA -3'	Regulator of virulence genes	55
ipgD	*ipgD*-F: 5'- GAGATTCCTATAAAGGCGCA -3'<br>*ipgD*-R: 5'- TTTTTGACGTATCACCACCT -3'	*Shigella* entry	55
icsA	*icsA*-F: 5'- TGGTGACTCTATTACCGGAT -3'<br>*icsA*-R: 5'- ATAACTATCGCCACCATGTC -3'	*Shigella* adherence	55
ptsG	*ptsG*-F: 5'- CGTTGTATCGCATGTTATGG -3'<br>*ptsG*-R: 5'- AGGTAAATGCAGTACCAGTG -3'	Glucose transporter	55
pflB	*pflB*-F: 5'- CTGTGGGACAAAGTAATGGA -3'<br>*pflB*-R: 5'- CATTTTGATACCACCGAACG -3'	Pyruvate formate lyase	55
pta	*pta*-F: 5'- CTGGATCGAATCTCTGACTG -3'<br>*pta*-R: 5'- ACCTAGTTCTACACCCTGAG -3'	Phosphate acetyltransferase	55
16s (rrsG)	*16s*-F: 5'- ACGTCAATGAGCAAAGGTAT -3'<br>*16s*-R: 5'- AGATGGGATTAGCTTGTTGG -3'	Ribosomal RNA; internal control	55

- For HeLa

Gene name	Sequence (5'→3')	Function	Tm
il8	*il8*-F: 5'- AGATGTCAGTGCATAAAGACA -3'<br>*il8*-R: 5'- TCAGCCCTCTTCAAAAACTT -3'	Chemokine	55
nod1	*nod1*-F: 5'- CTCACCCCCACATTCAATTA -3'<br>*nod1*-R: 5'- CAAGTAGAGGAAGAACTCGG -3'	Nod-like receptor	55
pi3k	*pi3k*-F: 5'- CCTGAACTCACATCAGTCAA -3'<br>*pi3k*-R: 5'- TGTAGTCTTTCCGAACTGTG -3'	Membrane remodeling	55
glut1	*glut1*-F: 5'- ATTACTCCACGAGCATCTTC -3'<br>*glut1*-R: 5'- CAGATAGGACATCCAGGGTA -3'	Glucose uptake	55
orai1	*orai1*-F: 5'- TCAAGTTCTTGCCCCTCAAG -3'<br>*orai1*-R: 5'- GTCGGTCAGTCTTATGGCTA -3'	Calcium transporter	55
gapdh	*gapdh*-F: 5'- CGAGATCCCTCCAAAATCAA -3'<br>*gapdh*-R: 5'- TCTTGAGGCTGTTGTCATAC -3'	Glyceraldehyde-3-phosphate dehydrogenase; internal control	55
18s (RNA18SN2)	*18s*-F: 5'- AAGCTCGTAGTTGGATCTTG -3'<br>*18s*-R: 5'- CAGTTCCGAAAACCAACAAA -3'	Ribosomal RNA; internal control	55

Table S2. The detailed information of the selected genes for major indicators involved in pathogen infection

- In *Shigella*

Gene	Function	Reference

virF, virulence regulon transcriptional activator	One of the main T3SS activators for activating the downstream T3SS-related virulence genes	(3)
ipgD, inositol phosphate phosphatase	One of the main T3SS effectors for inducing membrane ruffling and enhancing *Shigella* invasion	(4)
ptsG, glucose transport	One of the main glucose transporters which control glucose utilization in *Shigella* and its growth	(5)
pflB, pyruvate formate-lyase	The main enzyme for controlling formate production within glucose metabolism	(6)
pta, phosphate acetyltransferase	The main enzyme for controlling acetate production within glucose metabolism	(7)

- In HeLa

Gene	Function	Reference
il-8, interleukin-8	The key pro-inflammatory cytokine in host cells for regulating neutrophil recruitment in *Shigella*-induced colitis	(8)
nod1, nucleotide-binding oligomerization domain	One of the main cytosolic pattern recognition receptors stimulated with *Shigella* for inducing the downstream IL-8 secretion in host cells	(9)
pi3k, phosphoinositide 3-kinase	PI3K/Akt, one of the main signal transduction pathways for controlling the expression of glucose transporter and regulation of the downstream transcription factor nuclear factor κB (NF-κB) mediated inflammatory responses in host cells	(10, 11)
glut1, glucose transporter-1	One of the main glucose transporters in host cells	(10)
orai1, calcium channel protein-1	Calcium channel regulator mediated LPS-stimulated membrane ruffling in host cells during pathogen invasion	(12, 13)
gapdh, glyceraldehyde 3-phosphate dehydrogenase	A house-keeper enzyme mediating glycolysis in host cells generally used for the normalization but its expression level also can be up-regulated by the stimulation of LPS from pathogen	(14)

References

1. Lerche MH, Karlsson M, Ardenkjaer-Larsen JH, Jensen PR. 2019. Targeted Metabolomics with Quantitative Dissolution Dynamic Nuclear Polarization. Methods Mol Biol 2037:385-393.
2. Livak KJ, Schmittgen TD. 2001. Analysis of relative gene expression data using real-time quantitative PCR and the $2^{-\Delta\Delta CT}$ Method. Methods 25:402-8.
3. Di Martino ML, Falconi M, Micheli G, Colonna B, Prosseda G. 2016. The Multifaceted Activity of the VirF Regulatory Protein in the *Shigella* Lifestyle. Front Mol Biosci 3:61.
4. Mattock E, Blocker AJ. 2017. How Do the Virulence Factors of *Shigella* Work Together to Cause Disease? Front Cell Infect Microbiol 7:64.
5. Kentner D, Martano G, Callon M, Chiquet P, Brodmann M, Burton O, Wahlander A, Nanni P, Delmotte N, Grossmann J, Limenitakis J, Schlapbach R, Kiefer P, Vorholt JA, Hiller S, Bumann D. 2014. *Shigella* reroutes host cell central metabolism to obtain high-flux nutrient supply for vigorous intracellular growth. Proc Natl Acad Sci U S A 111:9929-34.
6. Koestler BJ, Fisher CR, Payne SM. 2018. Formate Promotes *Shigella* Intercellular Spread and Virulence Gene Expression. mBio 9:e01777-18.

7. Kim YR, Brinsmade SR, Yang Z, Escalante-Semerena J, Fierer J. 2006. Mutation of phosphotransacetylase but not isocitrate lyase reduces the virulence of *Salmonella enterica* serovar Typhimurium in mice. Infect Immun 74:2498-502.
8. Singer M, Sansonetti PJ. 2004. IL-8 is a key chemokine regulating neutrophil recruitment in a new mouse model of *Shigella*-induced colitis. J Immunol 173:4197-206.
9. Buchholz KR, Stephens RS. 2008. The cytosolic pattern recognition receptor NOD1 induces inflammatory interleukin-8 during *Chlamydia trachomatis* infection. Infect Immun 76:3150-5.
10. Beg M, Abdullah N, Thowfeik FS, Altorki NK, McGraw TE. 2017. Distinct Akt phosphorylation states are required for insulin regulated Glut4 and Glut1-mediated glucose uptake. Elife 6:e26896.
11. Ashida H, Mimuro H, Sasakawa C. 2015. *Shigella* manipulates host immune responses by delivering effector proteins with specific roles. Front Immunol 6:219.
12. Tran Van Nhieu G, Dupont G, Combettes L. 2018. Ca(2+) signals triggered by bacterial pathogens and microdomains. Biochim Biophys Acta Mol Cell Res 1865:1838-1845.
13. Lopez-Guerrero AM, Tomas-Martin P, Pascual-Caro C, Macartney T, Rojas-Fernandez A, Ball G, Alessi DR, Pozo-Guisado E, Martin-Romero FJ. 2017. Regulation of membrane ruffling by polarized STIM1 and ORAI1 in cortactin-rich domains. Sci Rep 7:383.
14. Xie W, Shao N, Ma X, Ling B, Wei Y, Ding Q, Yang G, Liu N, Wang H, Chen K. 2006. Bacterial endotoxin lipopolysaccharide induces up-regulation of glyceraldehyde-3-phosphate dehydrogenase in rat liver and lungs. Life Sci 79:1820-7.
